# Rule-based multi-level modeling of cell biological systems

**DOI:** 10.1186/1752-0509-5-166

**Published:** 2011-10-17

**Authors:** Carsten Maus, Stefan Rybacki, Adelinde M Uhrmacher

**Affiliations:** 1 University of Rostock, Institute of Computer Science, Albert-Einstein-Str. 22, 18059 Rostock, Germany

## Abstract

**Background:**

Proteins, individual cells, and cell populations denote different levels of an organizational hierarchy, each of which with its own dynamics. Multi-level modeling is concerned with describing a system at these different levels and relating their dynamics. Rule-based modeling has increasingly attracted attention due to enabling a concise and compact description of biochemical systems. In addition, it allows different methods for model analysis, since more than one semantics can be defined for the same syntax.

**Results:**

Multi-level modeling implies the hierarchical nesting of model entities and explicit support for downward and upward causation between different levels. Concepts to support multi-level modeling in a rule-based language are identified. To those belong rule schemata, hierarchical nesting of species, assigning attributes and solutions to species at each level and preserving content of nested species while applying rules. Further necessities are the ability to apply rules and flexibly define reaction rate kinetics and constraints on nested species as well as species that are nested within others. An example model is presented that analyses the interplay of an intracellular control circuit with states at cell level, its relation to cell division, and connections to intercellular communication within a population of cells. The example is described in ML-Rules - a rule-based multi-level approach that has been realized within the plug-in-based modeling and simulation framework JAMES II.

**Conclusions:**

Rule-based languages are a suitable starting point for developing a concise and compact language for multi-level modeling of cell biological systems. The combination of nesting species, assigning attributes, and constraining reactions according to these attributes is crucial in achieving the desired expressiveness. Rule schemata allow a concise and compact description of complex models. As a result, the presented approach facilitates developing and maintaining multi-level models that, for instance, interrelate intracellular and intercellular dynamics.

## Background

In computational modeling of cell biological processes, a formal representation, i.e. a model, of the dynamics of the system under study is the central subject of investigations. Cell biological models typically focus on the processes of molecules like proteins and small chemicals. However, in addition, dynamics at cell level, e.g. proliferation and differentiation of stem cells, and cell-cell interaction, influence these intracellular dynamics as well, just like such high-level dynamics are influenced by processes at the molecular level. This hierarchical organization and the causalities between different levels, i.e. from the lower to the upper (upward causation) and vice versa (downward causation), are universal characteristics of biological systems [1,2]. Hence, multi-levelness has been identified to be an important and general principle of systems biology [3]. Depending on the question that shall be pursued with the model, capturing processes that happen at different levels, e.g. proteins, individual cells, and cell populations, and their interrelations within the model is of relevance [4]. The question is how can this multi-levelness be supported by modeling methodologies? We will pursue this question in the context of rule-based modeling.

### Rule-based modeling

In the past years, many different modeling languages have been introduced to support modelers in their task, for example [5-8]. The idea is to write down a model not directly mathematically, like in ordinary differential equations (ODEs) or stochastic processes, but in terms of a tailor-made syntax. A semantics is then provided that bridges the gap between what is written and the mathematical definition of its computation. A carefully designed syntax can increase the accessibility of models for discussion and presentation, especially for domain experts that are not extensively familiar with modeling and the underlying mathematical formalism. Formal modeling languages can also extend the flexibility in the choice of methods for model analysis, since more than one semantics can be defined for the same syntax (see [9-12] for some examples).

Rule-based modeling languages use the notation of chemical reaction equations (or very similar representations), which denote a natural choice of syntax to model cell biological systems. Consider, for example, a simple reversible process of dimerization as it occurs in many signaling pathways [13,14]. It can be described by the two chemical species *Monomer *and *Dimer *and the following reversible reaction, where *k*_*f *_and *k*_*r *_are the respective rate constants for the forward and backward reactions:

$$Monomer+Monomer\overset{k_{f}}{\underset{k_{r}}{⇌}}Dimer$$

Chemical solutions, i.e. mappings from species to concentrations or alternatively to their discrete integer amounts, describe a model's state. A formal semantics can then be defined as mapping of chemical solutions and reactions to, for example, stochastic processes or ODEs [11,15].

Many rule-based approaches, for instance [15-17], allow to describe species with attributes as well as rules with reactant patterns, i.e. by specifying structured molecules and rule schemata they allow to model basic reactions as an alternative to the entire network of all possible chemical species and reactions. Thus, due to rule schemata, complexity - in terms of the number of required rules - can be significantly reduced [18]. 

Let us illustrate this with the help of a simple example. Proteins, in particular those involved in signaling pathways, often show various sites for binding other molecules. Furthermore, modifications like phosphorylation or methylation at diverse sites might determine the ability for binding other molecules and thus influence the interaction pattern of a protein. Hence, combinatorial explosion easily leads to very complex network models with hundreds or even thousands of species and reactions. Consider a system of ten interacting proteins. One of them is a large scaffolding protein that might reversibly bind each of the other nine proteins at nine distinct sites. We assume each of the nine binding reactions to be independent from other bindings. This at first view quite simple model requires to define 521 (2^n ^+ *n *with *n *= 9) molecular species, i.e. distinct combinations of bindings, and 4608 reactions. Attributes and rule schemata help to deal with the system's complexity by specifying only the basic molecules and reactions. As the binding reactions are assumed to be independent from each other, each of them may be described individually without taking the state of other binding sites into account. By omitting such irrelevant information, one rule might then be translated into multiple basic reactions of a large network by which the model is kept small and manageable. Hence, a rule-based modeling language like BioNetGen [11] allows to model the above system by specifying a set of only ten molecules and nine reversible rule schemata instead of 521 molecular species and 4608 reactions. For a more comprehensive review of rule-based modeling and its advantages for formal descriptions of signal transduction pathways, we would like to refer to [18].

Summing up, due to schematic rules, the complexity of a model may be effectively reduced and an intuitive modeling metaphor along the lines of well-known chemical reaction equations facilitates the process of modeling and the accessibility of models. Consequently, the number of rule-based approaches to describe biochemical reactions has increased during the last years, e.g. [8,11,15,16], and also an increasing number of publications can be observed that utilize rule-based languages for concrete modeling studies, e.g. [19-22]. 

In this paper, we identify concepts for supporting rule-based multi-level modeling. We show a realization of these concepts as part of ML-Rules, a modeling and simulation approach we developed. Thereby, we start with concepts that nearly all current state of the art rule-based approaches support to successively approach concepts that are obviously related to multi-levelness, i.e. nesting. Thereafter, an example in which intracellular and intercellular dynamics are combined will illuminate the role that each of these concepts play in supporting multi-level modeling. Finally, to complete the results and discussion part of the paper, related work will be revisited to discuss which of the identified concepts are already supported.

## Results and Discussion

### Overview of Concepts

A very brief introduction to rule-based modeling is already given in the previous background section. In the following, we will focus on the concepts we use in our rule-based multi-level approach (ML-Rules). Their respective role in supporting multi-level modeling will be shown in the subsequent example model. 

For first studies, we base ML-Rules on continuous time Markov chains (CTMCs). The semantics is discrete population-based, i.e. we work with natural copy numbers of identical species instead of real valued concentrations. The reason for a stochastic semantics lies in the observation that at higher levels of organization (like cells) no longer abundant numbers will be able to balance fluctuations as can be often observed at lower levels, e.g. proteins that are involved in metabolic pathways. And also when looking at levels further down in the hierarchy, e.g. gene regulatory processes, stochastic events may play a crucial role due to low copy numbers of involved species. Hence, stochasticity is often an essential feature for multi-level modeling of cell biological systems [23]. However, it should be noted that stochasticity is not necessarily constrained to CTMCs, as sometimes at higher levels other than exponential time delays are required, e.g. normal distributions [24].

#### Species, attributes, and solutions

The basic building blocks of ML-Rules models are called species which may represent any object of interest, e.g. small chemicals, macro-molecules like proteins, or membrane bound cellular compartments. Each species has a name, e.g. *A*, and each name has a fixed arity *ar *∈ ℕ_0 _that specifies the number of attributes of a species. Attributes are not restricted to a finite set of values and they may be of any kind of numerical value and textual string. For convention throughout this paper, species names start with a capital letter and attributes are written in a bold font type within parentheses behind the name. Parentheses are omitted if *ar *= 0. For example, *A, A*(**1**), *A*(**0**,**1.67**), and *A*(**green**,**-15**,** true**) are valid examples for a species *A *with *ar*(*A*) = 0, 1, 2, and 3 respectively. However, these examples are invalid when two or more of them are being used within the same model, as the arity of a species name is fixed and therefore may not vary between species with identical names, i.e. *A *in this case. Each defined combination of attributes is a distinct species, i.e. *A*(**1**,** 1**) and *A*(**1**,** 2**) share the same name but are different species.

A solution is a multiset of species, i.e. can be either a single species or a composition of multiple sub-solutions. The '+' is the delimiter symbol for composing multiple solutions and a solution can be also an empty set ∅. We write *nA *with *n *∈ ℕ_0 _to refer to a solution which is composed of *n *identical copies of *A *(*n *is omitted if *n = *1). For example, [2 A(**1**) + 4A(**2**) + B] describes a solution consisting of three different species with an amount of 2, 4, and 1 respectively.

#### Reaction rules, rule schemata, and their instantiation

Reaction rules describe the dynamics of a model, i.e. they define how certain species are removed from or added to a given solution. When firing, a rule substitutes a reactant solution *S *by a product solution *S'. *The general syntax follows the notation of chemical reaction equations and the majority of other rule-based modeling languages, e.g. [8,9,16,17,25], namely reactants are written on the left-hand side and products on the right-hand side of an intermediate right-headed arrow:

$$S\to S^{\prime }.$$

For simplicity reasons, our syntax only allows for uni-directional rules. Thus, two complementary rules have to be defined for modeling reversible reactions. However, it would be straightforward to extend the syntax to reversible reaction rules if needed.

Rule schemata are a notational convenience, which uses variables to bind attributes of reactants. By doing so, each rule schema may encode for several rule instantiations, i.e. reactions. Let us take a reaction which converts a species *A *into another species *B. *Consider the situation, that *A *is an attributed species with an arity *ar*(*A*) = 1, but the attribute is of no interest for its reaction to *B*. Without schematic rules, we would need to specify one rule for each possible value of the attribute of *A*, which can be tedious and error-prone.

Moreover, as we do not fix the set of attribute values, i.e. the state space might be infinite, it is impossible to define each potential reaction. Therefore, instead of specifying rules with the defined reactant species, we define a reactant *pattern *by inserting a variable *x *for the attribute of *A*:

A(x)→B.

For convention, we write variables in a non-bold font type and starting with a lower case letter throughout this paper. Mapping the above rule schema to the solution [2 *A*(**1**) + 4*A*(**2**)] evaluates to the following two rule instantiations:

A(1)→B and A(2)→B.

Besides such very simple variants, rule schemata can be also defined by employing expressions to specify attributes. For instance, a reactant pattern *A*(*x*) + *A*(2*x*) matches every solution where at least two *As *exist, one of which attribute's value is exactly twice the attribute's value of the other one. Expressions can be also used to specify the attribute of the products, e.g. the rule *A*(*x*) → *B*(2*x*) applied to a solution [*A*(**2**) + *B*(**3**)] would lead to [*B*(**3**) + *B*(**4**)]. Please note, arbitrary functions can be used for expressions (see also the section on implementation).

A further reduction in the number of rules can be achieved by using attributes to represent links between species, e.g. to model noncovalent bonds within protein complexes. By doing so, individual subunits of a protein complex can be preserved instead of specifying numerous species names, each of which would reflect a different combination of subunit states and bindings (recall the scaffold protein example in the background). Entirely new values can be created with the help of the *ν*-operator:

A(F)+B(F)→(νx)A(x)+B(x).

**F **is a constant that denotes a free binding site and *νx *creates a fingerprint-like unique value which does not already occur in the current model state. It is assigned to the products on the right hand side of the rule via variable *x, *i.e. in an instantiation of the rule, *x *is replaced by a newly created unique value which serves as identifier for this particular binding. This method for representing linkage of species is identical to private channels in the π-calculus [6,26] and allows to model molecular complexes similar as can be done with rule-based languages that have explicit notions of complexation, e.g. [16,17]. Moreover, once created, unique values can be used in a highly flexible manner, e.g. to describe bonds shared by more than two binding partners (like hyperedges in a graph) or across level boundaries (the concept of multiple levels will be introduced below). Species can also be marked with an unique indentifier to observe the dynamics of individual entities.

However, note that the approach also has some drawbacks compared to explicit notions of molecular bindings: first of all, without profound knowledge or decent annotation of the model, it might be difficult to find out whether certain attributes of species represent binding sites. The approach would also allow to reset just one species to its unbound state while its former binding partner remains unchanged. Therefore, the modeler is responsible for describing a correct model without such unrealistic dynamics. Furthermore, at least in the current implementation of ML-Rules, using identifiers for modeling links between species may slow down the simulation as the number of distinct species increases and therefore matching reactants may take significantly more time (see section on implementation).

#### Kinetic rates and constraints

Each reaction rule is assigned a stochastic kinetic rate $r\in {\mathbb{R}}_{0}^{+}$:

$$S\overset{r}{\to }S^{\prime }.$$

The higher the rate of a rule, the more likely the rule will fire at a time calculated according to this rate. The kinetic rate can be a simple constant numerical value, for example, to describe a chemical reaction with constant speed, i.e. a zeroth-order reaction whose speed is independent of the amount of any chemical species. However, most reaction rules that describe biological systems need to take the amount of one or more reactant species into account for specifying correct system dynamics. Probably in most cases the kinetics of a rule follows the law of mass action, but in systems biology alternative kinetics, e.g. Michaelis-Menten kinetics for enzymatic reactions and Hill functions for describing cooperativity, are also frequently applied [27]. That is why we allow for arbitrary reaction rates using mathematical expressions. Any kind of mathematical expression is allowed that evaluates to a non-negative numerical value, see also [28].

Species identifier are used to refer to the amount of species in a given solution. We assign reactant *A *a species identifier *a*, i.e. *A*(...)^*a*^, which evaluates to the amount of species *A*(...) in the solution. Assuming mass action kinetics, the rate of a first-order reaction *A *→ *B *with rate constant *k *can then be correctly described as follows:

$$A{\left(x\right)}^{a}\overset{k.a}{\to }B.$$

The evaluation of its mapping to the solution [2*A*(**1**) + 4*A*(**2**)] leads to two rule instantiations with different propensities:

$$A\left(1\right)\overset{k.2}{\to }B\;\text{and}\;A\left(2\right)\overset{k.4}{\to }B.$$

Like in the *attributed π-calculus *[29] and React(C) [15], reaction constraints allow for more powerful control on the dynamics of a model.

For example, we can constrain the preceding rule to only fire when the amount of *A*(*x*) exceeded a certain threshold value *T:*

$$A{\left(x\right)}^{a}\overset{\text{if}\;a>T\;\text{then}\;k.a\;\text{else}\;0}{\underset{}{\to }}B.$$

If the amount of *A*(*x*) in a given solution does not exceed *T*, the if-then-else expression evaluates to a kinetic rate of 0, which determines that the rule will not fire.

To enhance the readability of rules with such constraints, we use an extra notation. Instead of a complex rate consisting of the expression if *e *then *r *else 0, we write the conditional expression *e *below the arrow that is assigned the basic kinetic rate *r*:

$$S\overset{r}{\underset{e}{\to }}S^{\prime }≜S\overset{\text{if}\;e\;\text{then}\;r\;\text{else}\;0}{\to }S^{\prime }$$

The preceding example now looks as follows:

$$A{\left(x\right)}^{a}\underset{a>T}{\overset{k.a}{\to }}B.$$

#### Multi-level rule schemata

The features listed so far are not new. Species with attributes and schematic rules are standard features that can be found in nearly every rule-based language in the field of systems biology, e.g. [11,15,16]. The requirement for modeling biological systems with rate kinetics different from those following the law of mass action seems to be also widely accepted nowadays. BIOCHAM [9], LBS [8], and React(C) [15], for example, support arbitrary reaction rates. Also recent developments for the BioNetGen language now allow to specify user-defined rate law functions, and moreover, modeling of conditional expressions to support the construction of logical sequences of control [30]. All together, they will play a role in supporting multi-level modeling.

However, a truly salient feature of multi-level modeling are hierarchies. Hierarchical structuring facilitates modeling of complex biological systems by defining them in terms of their components and the interactions that exist between them. Hierarchies help to structure the knowledge about a given system [31]. In addition, they allow to describe multiple nested solutions similar to the multiple separated reaction compartments that can be found in biological systems, e.g. cells, organelles, and vesicles. 

To address the need for hierarchical model structures, we introduce the concept of nested species. That means species may not only be characterized by names and their attributes, but also by a potentially enclosed solution of further species. Let us give an example where *A*, *B*, *C*, *D*, and E denote different names of species. Solution S consists of two species *A *and *B *of which both contain solutions with further species on their own. Species *A *consists of a sub-solution *S*_*A *_= [C + 2 *D*[*S*_*D*_]], i.e. a single atomic species *C *and a nested species of type *D*[*S*_*D*_] with an amount of two and a sub-solution *S*_*D *_= [*E*]. Equally to S_D_, the sub-solution enclosed by *B *consists also of species with name *E*, but here with an amount of three: *S*_*B *_= [3*E*]. The whole nested solution can be written as:

$$S=\left[A\left[C+2\;D\right)\left[E\right]\right]+B\left(\left[3E\right]\right].$$

**Figure 1:**
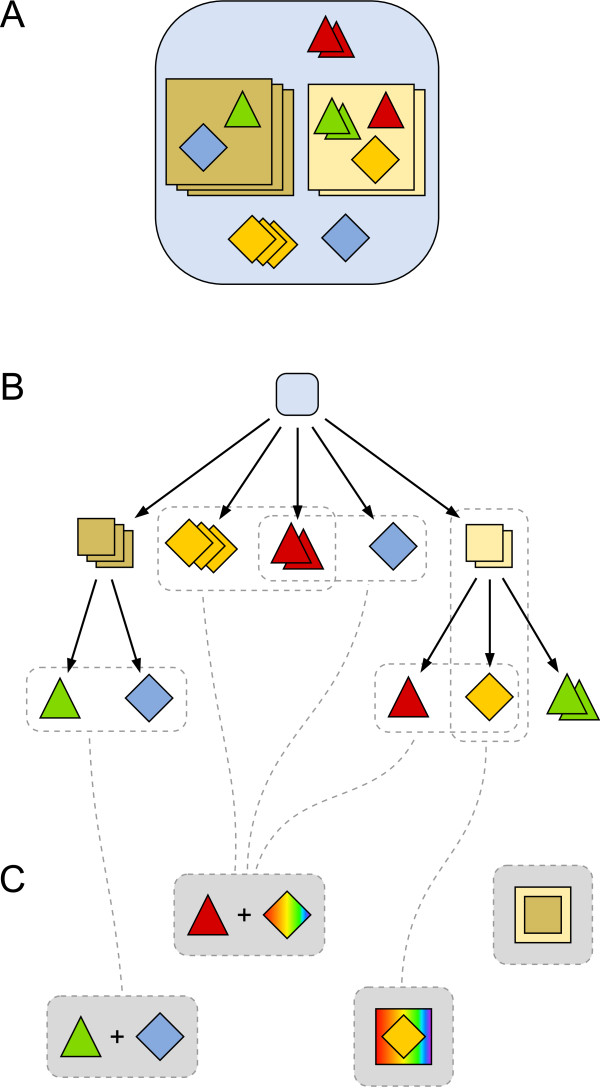
**Nested model structure**. Illustration of the hierarchical modeling concept. Different shapes of nodes correspond to different species names while attributes are color-coded. Stacking of identical nodes depicts the amount of a certain species. (A) Graphical representation of a hierarchical model structure via nested nodes. (B) The same model structure alternatively depicted as a directed tree graph. Please note that besides atomic species (triangles and diamonds) also species containing a sub-solution (squares) might be attributed so that each species at each level might has its own state. (C) Examples of matching different reactant patterns within the hierarchical model structure. The rainbow shadings in the second and third pattern illustrate variable instead of defined colors, i.e. attributes.

To avoid confusion by too many brackets and to get a quick visual impression of the nesting, in the following we will use a graphical representation of nested nodes:

Please note that nested species may still have assigned attributes (see also Figure 1A and 1B). In the textual syntax, attributes and the enclosed solution are embraced by different kinds of brackets, i.e. *A*(**0**)[*S*_*A*_] denotes the existence of an attribute 0 for the above nested species *A*. The graphical syntax is straightforward:

The ability to assign attributes to nested species allows to equip each hierarchical level with an own state that is not only determined by the enclosed species. Such high-level states are of particular interest for multi-level modeling as they allow to describe dynamic behavior similar to observations performed at different levels of organization. The later example model will illustrate this in detail.

**Figure 2:**
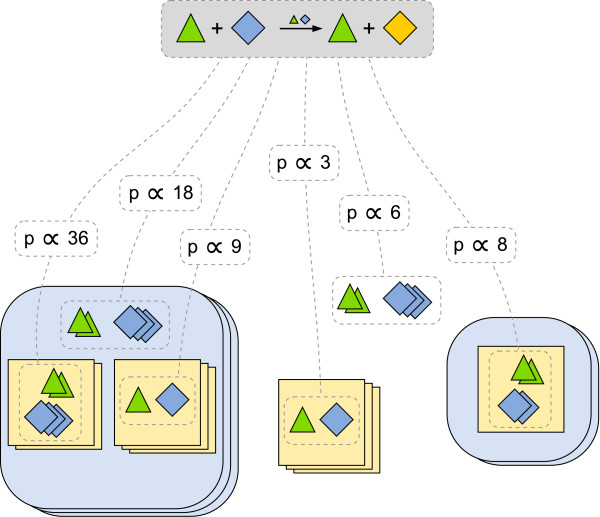
**Hierarchy-dependent instantiation of rules**. Due to population-based aggregation of species, the propensity (firing rate) of a rule instantiation is not only determined by the actual rate of a schematic rule (here the amount of green triangles and blue diamonds) but it is also context dependent, i.e. it is proportional to the amount of enclosing species. Therefore, enumeration of rule instantiations requires to calculate the propensity p dependent on the whole hierarchy above the matched reactant species. For example, the propensity of the rightmost matching is proportional to 2·2·1·2 = 8, where the first two factors correspond to the amount of triangles and diamonds, the third describes the single enclosing rectangle, and the last factor corresponds to the amount of the outermost enclosing species (light blue with round corners).

A nested hierarchical model structure opens the door for reducing model complexity not only by specifying rule schemata as described above, i.e. by specifying reactant species with variables instead of defined attribute values. The number of rules needed may be also reduced by applying rules to multiple solutions, so that reactants can be matched at different levels and within solutions enclosed by different species types (Figure 1C). However, this has an important consequence for the semantics. When applying rules to solutions and calculating the propensity of a reaction, one needs to take the context of this application into account, which is given by the amount of species at higher levels (Figure 2). The propensity of the rule has to be adjusted according to the whole hierarchy above, as a reaction is more likely to happen the more solutions exist it could potentially take place in.

To let different levels of a hierarchical model interact with each other, a rule may involve nested reactants and/or products. Such rules look pretty much the same as rules of flat models do. In principle, also the enumeration of such rules works similar. For example, the rule

describes a reaction from *A*(**0**) to *A*(**1**) under the condition that *A *encloses at least one species *C. *Please note, this rule may also match species where *A*(**0**) contains further species in addition to the *C *(no matter which and how many), for instance like in the previous example. However, such a sub-solution gets lost when the rule fires, as the product species contains just exactly one *C*. The same holds true for a potential sub-solution of *C*; the reactant pattern matches every species where a *C *is part of a sub-solution of *A*(**0**), but it says nothing about a sub-solution of *C. *Hence, if the reactant species *C *would contain further species, they would get lost as well.

To prevent this from happening, we would need to specify this explicitly, by binding the solution to the variable *x, *defining a guard for the reaction, i.e. *C *∈ *x, *and inserting *x *into the product:

$$A(0){\left[x\right]}^{a}\underset{C\in x}{\overset{k.a}{\to }}A(1)\left[x\right].$$

As it is often the case that we want to preserve the rest of the solution, we provide a specific rule schema for this case where a variable binds to the entire rest of a solution (the dashed rectangle in the following example) and can be used to reinsert this solution on the product side of the rule. The above rule can be specified now as:

Such bound solutions can be freely reused for defining the products, i.e. migration, copying, and merging of solutions are easy tasks. The problem of splitting is another matter, for which specific operations on solutions are needed, e.g. to split a solution equally into two new solutions. Whereas ML-Rules and its current simulator allow the use of arbitrary functions on attributes, the application of functions on solutions is not yet supported. However, an integration into ML-Rules requires only slight adaptations of the syntax and semantics and is therefore planned for the near future.

Later, we will provide a more detailed explanation of our multi-level approach based on a realistic biological system. With the help of this example, we will also motivate again the need for multi-level modeling and illustrate how to realize upward and downward causation.

#### Implementation

**Figure 3:**
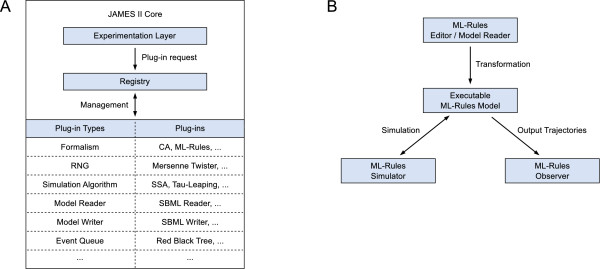
**Architecture of ML-Rules and the JAMES II framework**. (A) Basic overview of the JAMES II modeling and simulation framework architecture. The core defines a basic set of plug-in types and plug-ins needed to run experiments and also provides a rich set of tools reusable in other plug-ins. Also part of the core, the registry is responsible for managing plug-in types and plug-ins, and the experimentation layer carries out simulation experiments, e.g. simple simulation runs, parameter scans, optimizations and sensitivity analyses. (B) Simplified overview of the main ML-Rules plug-ins and how they are interconnected. Arrows show flow of data.

The modeling and simulation environment for ML-Rules has been realized within the modeling and simulation framework JAMES II [32]. Figure 3A gives a very brief overview of the JAMES II framework, which consists of a *core *and a large set of different *plug-ins. *For ML-Rules, a set of new plug-ins has been implemented: an editor that allows to create and edit ML-Rules models supporting syntax highlighting (including syntactical and semantical consistency checks) and a simulator which is based on the Direct Reaction Method of Gillespie [33] and thus implements an exact stochastic simulation algorithm (SSA). In addition, plug-ins for model reading and writing and for observing the model have been realized (see also Figure 3B). These are the typical plug-ins that have to be implemented if a new formalism shall be added to JAMES II. Other plug-ins can simply be reused, e.g. for random number generation and event queues. Also plug-ins for (parallel) optimization, validation, trace analysis, data storage, etc. can be reused to support the execution of entire simulation studies [34].

Below we provide a basic description of the simulation algorithm. Please note that in JAMES II simulation algorithms are not designed as monolithic blocks. By using plug-ins, alternative sub-algorithms can be easily exploited and combined. It has been shown that the performance and suitability of algorithms depend to a large degree on the concrete model, that details (i.e. sub-algorithms) matter, and that a suitable configuration can significantly speed up simulation [35]. In combination with methods that help to automatically select and configure simulators on demand, this type of simulation design supports a high flexibility for executing multi-level models. Therefore, the simulator is structured as follows.

**Require**: *S, rules*

   **for ***ru *∈ *rules ***do**

      *rts *← *MatchReactants(ru*, *S)*

      *rcts *← *rcts *| *CreateReactions*(*ru*, *rts*, *S*)

   **end for**

   **for **r ∈ *rcts ***do**

      *r*_*prop *_← *CalcPropensity*(*r*)

   **end for**

   *reaction *← *SSA(rcts)*

   **for ***reactant *∈ *reaction ***do**

      *RemoveReactant*(*reactant*)

   **end for**

   **for ***product *∈ *reaction ***do**

      *PutProduct*(*product*)

   **end for**

*MatchReactants *selects all matching reactants of the selected rule schema *ru. *The next step is to instantiate the rule schema *ru *and grouping equivalent rule instances by calculating the reactions *rtcs. *Now the propensity is calculated for each reaction *r*. Please note, as now the number of reactants (that apply) is part of the reaction, calculating the propensity is only dependent on *r*. Also, all information needed is directly available for each *product *in *r*, such as bound attribute values or solutions that are used on the rule's product side. After that an SSA is invoked. We have so far integrated the Direct Reaction Method of Gillespie. The selected reaction is executed by removing reactants and adding products. 

The complexity of *MatchReactants *for matching a reactant is $O\left(n+m^{k}\right)$ where *n *denotes the number of species in the solution, *m *the number of species in one context and *k *the depth of nesting of the reactant. The complexity of *CreateReactions *is $O\left(l^{n}\right)$ where l is the number of reactants of a rule. Some optimizations are employed, e.g. to restrict the search space for matching reactants. For example, when evaluating a rule *A*(**4**) → *B, *all *A*s whose attribute does not equal 4 will not be considered for matching the reactants of a rule to a solution. However, most of the simulation efforts still goes into calculating *rts*, i.e. matching the rules (coarsely 50% of the overall calculation). Therefore, current efforts are dedicated towards developing alternative approaches for matching, e.g. integrating special index methods. To avoid time-consuming instantiation of all possible reactions, an alternative kinetic Monte Carlo simulation approach [30,36,37] based on individual particles rather than populations of identical species, might also be worth to explore for simulating ML-Rules models.

With *CalcPropensity *the propensity of each generated reaction is calculated using the specified expression and taking the context of the matched solution into account. This means that the propensity is adjusted according to the amount of possible contexts the matched solution is part of (see previous Section and Figure 2). Based on Java reflection, currently functions provided by Java can be used within the expressions. The integration of a library of own functions as a plug-in will be realized in the future.

Maintaining the consistency of populations when executing *RemoveReactant *and *PutProduct *is a crucial part during simulation and requires, given the nested species, special attention. This means whenever a species s is removed from a solution *S*_*sub *_from the overall solution *S*, the populations within *S *need to be updated accordingly. Sometimes it might not be enough to just decrease the population value of *s *in *S*_*sub*_, because by removing s from *S*_*sub*_, *S*_*sub *_becomes a different species which means it needs to be split from the previous population it was attached to and needs to be merged with an already existing population of that species (see example below). This actually has to be carried on upwards the hierarchy until no splitting and merging is needed anymore. The following example shows splitting and merging. Given a solution:

$$S=\left[2A\left[2B\right]+2A\left[3B\right]\right].$$

Removing one *B *from *A*[3*B*], would first lead to a split of the population of *A*[3*B*] and result in the solution

$$S=\left[2A\left[2B\right]+A\left[3B\right]+A\left[2B\right]\right].$$

where *A*[2*B*] has to be merged with the already existing population of 2*A*[2*B*], so the correct solution will be

$$S=\left[3A\left[2B\right]+A\left[3B\right]\right].$$

The current simulator proceeds basically as a discrete event simulator. Therefore, only slight adaptations are required to support also events that are not distributed exponentially. This feature is important as many biological phenomena at higher levels are not necessarily exponentially distributed, as argued in [38]. Also, as multi-level models operate often at different temporal scales, the calculation effort for simulating these models in a pure discrete event manner might easily become prohibitive. Therefore, hybrid simulation approaches, e.g. [23,39], shall be exploited in the future.

With additional file 1 we provide a prototypical demo software tool comprising a model editor, simulator, a rudimentary line chart visualization, and simulation data export. The following examples as well as additional example models can be loaded and demonstrate the concrete syntax of ML-Rules. Its source code will be made available under an open source licence as part of a following JAMES II release at http://www.jamesii.org.

### Example model

**Figure 4:**
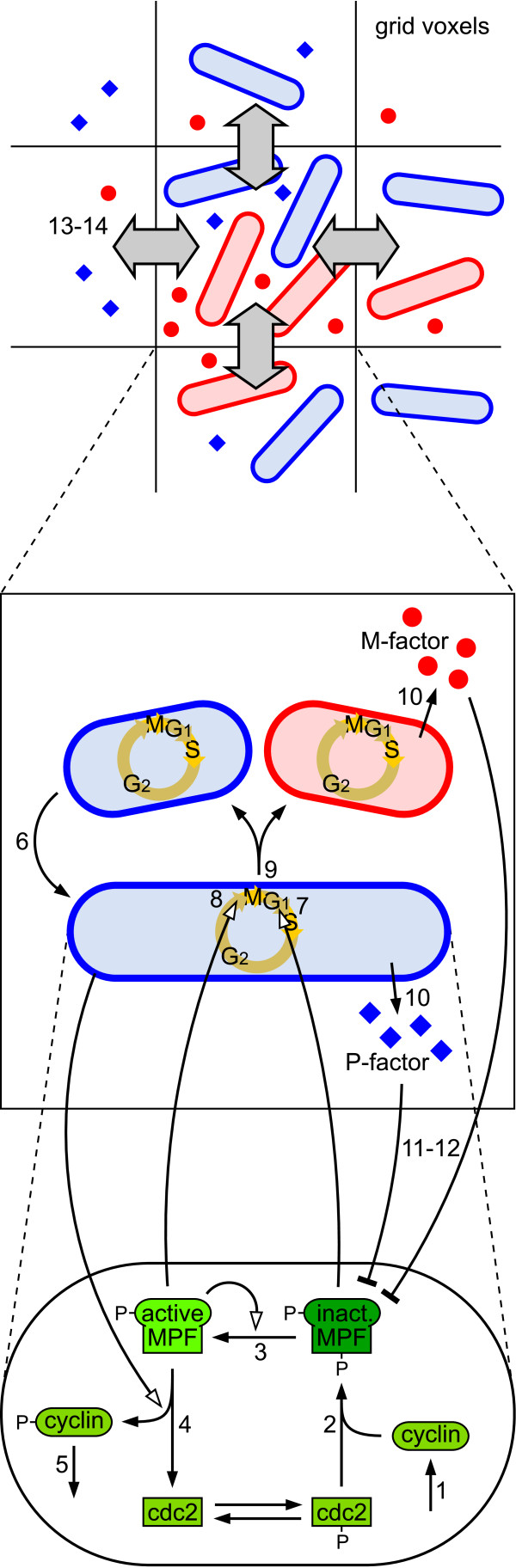
**Schematic description of the example model**. The example model comprises three distinct hierarchical levels. At the bottom level, interacting proteins describe the inctracellular dynamics of a fission yeast cell (reactions 1-5). The molecular species and reactions are similar to those described in [46]. The intermediate level describes dynamics of entire cell states, i.e. cell growth (6), cell cycle phase transitions (7-9), and division including mating type switching (9). In addition, cells may secrete pheromone molecules (P-factor and M-factor) to the extracellular medium (10). Various inter-level causalities between the intermediate and the bottom level influence processes both in an upward (7-9) and downward causation manner (4,11-12). The top level discretizes the environment of cells into multiple fictive compartments in order to study spatial dynamics of pheromone diffusion and displacement of cells (13-14). Abbreviations used for naming the species in the model: *Y *(cyclin), *Y*_*P *_(phosphorylated cyclin), *D *(cdc2), *M*_*I *_(inactive MPF), *M*_*A *_(active MPF), *C *(fission yeast cell), *F*_*P *_(P-factor pheromone), *F*_*M *_(M-factor pheromone), *G *(voxel of spatial grid).

We would like to motivate and illustrate our multi-level approach with an abstract multicellular model of the fission yeast (*Schizosaccharomyces pombe*) cell division and mating type switching in dependence of an intracellular control circuit. This intracellular regulatory network of interacting proteins in turn depends on the size of the cell and specific pheromone molecules. To prepare for mating, fission yeast cells may secrete pheromones that cause an arrest of the division cycle of cells with opposite mating type. So, the different parts of the model at multiple levels are highly interconnected and influence each other in various ways (see Figure 4). In addition, to investigate the relation between pheromone signaling and the location of cells, the model comprises also some simple spatial dynamics that cover pheromone diffusion and cell displacement from crowded areas.

The presented model illuminates the importance to consider different levels of organization for modeling certain phenomena in cell biology and shows the previously introduced concepts at work. Particularly the extension of the model from a single cell to multiple cells illuminates the benefits of the presented rule-based multi-level approach.

As the model is not intended to be a contribution for fission yeast science, it may not be sound in each aspect and may not reflect the current level of knowledge about this system. Also certain parameters are simply estimated. However, we payed attention to presenting a realistic case study that shows how a rule-based multi-level approach like ML-Rules facilitates modeling of such systems.

#### Cell division cycle

The eurkaryotic cell cycle consists of four distinct phases: G_1_, S, G_2_, and M. During the first three phases, a cell is increasing in size and its DNA is replicated. At the end of the cycle, a cell enters the M phase (mitosis) and finally divides into two daughter (or sibling) cells. These major events of the cell division cycle are controled by certain proteins and the underlying regulation processes in fission yeast have been extensively studied [40-45].

In our example model, the regulation at protein level is based on an early model by Tyson [46]. This deterministic continuous model consists of two proteins, cyclin and cdc2, that form a complex called maturation promoting factor (MPF) which in turn controls traversion through the cell cycle. Today, there exist much more detailed models of this system than the relatively simple Tyson model, e.g. [43,47-49]. However, the purpose here is not to provide the most accurate model of yeast cell cycle control but to show why multi-level modeling is important for studying certain aspects of cell division and how it can be realized. In this sense, the Tyson model is well suited as it is simple but at the same time captures the essential dynamics.

Most reactions of this model follow the law of mass action and we do not discuss each rule in detail here. Instead, we would like to refer to the supplementary material where the whole model can be found (additional file 2). The interesting reactions from our point of view are the activation of MPF and the subsequent dissociation of this complex. Activation of inactive MPF, i.e. the dephosphorylation of its cdc2 subunit, is assumed to be an autocatalytic process:

The higher the amount of activated MPF (*M*_*A*_), the higher is the activation rate; *D*_*tot *_is a model parameter that denotes the total amount of cdc2.

**Figure 5:**
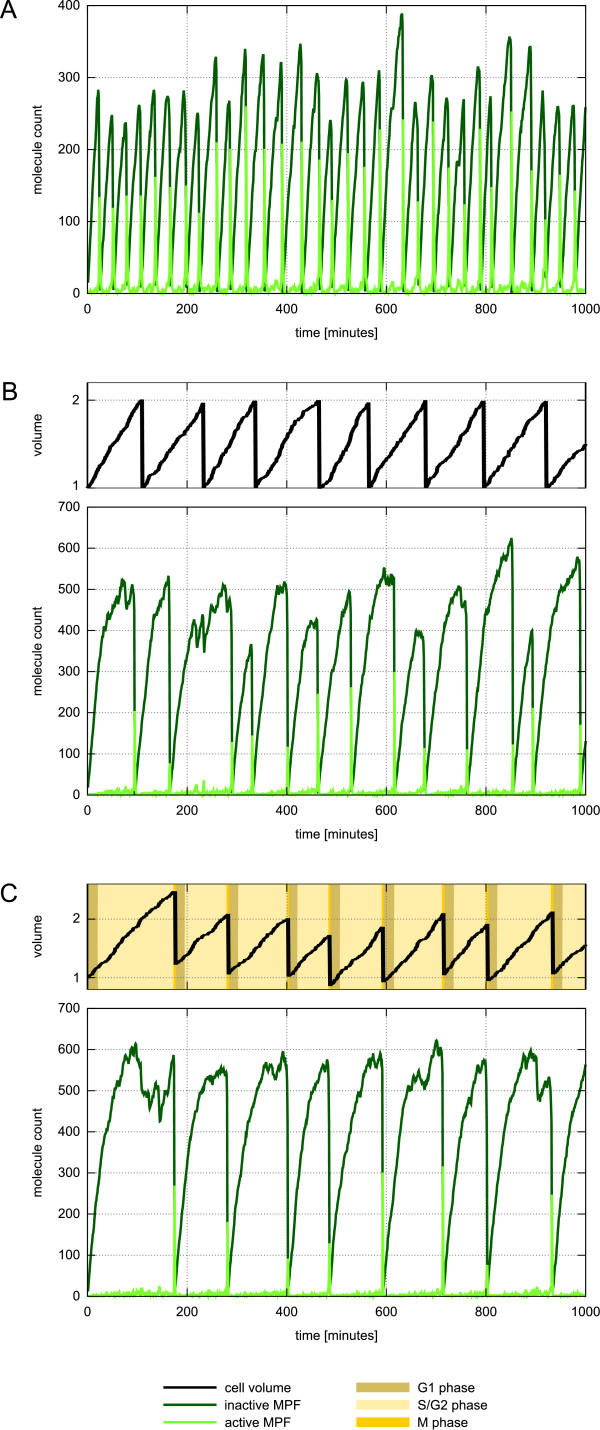
**Cell cycle dynamics of an individual fission yeast cell**. Simulation results of three different cell cycle models. Inactive MPF is depicted by dark green curves and light green denotes activated MPF. (A) Stochastic variant of the cell cycle model presented in [46]. Simulation parameters are similar to the parameters that have been used to produce Figure 3a in [46], i.e. *k*_3 _= 180 min^-1 ^and *k*_4 _= 0.9 min^-1^. (B) Model includes downward causation by dynamic adjustment of the MPF dissociation rate due to an increase of the cell volume. Parameters are equal to those presented for model 1 in the additional file 2. (C) Multi-level model comprising of downward and upward causation. MPF dissociation depends on the cell volume and at the same time, transitions from one cell cycle phase to another depends on the intracellular amount of active and inactive MPF. The entire model as well as initial solution and parameters can be found in additional file 2, model 1.

Tyson identified a region for two parameters (the rate constants for autocatalytic MPF activation and its dissociation) where regular cycle oscillations with bursts of the amount of the inactive and activated MPF complex can be observed. Although comprising fluctuations due to the stochastic processes, the intracellular reactions of our example model show similar oscillatory behavior (Figure 5A). The period of roughly 30 minutes between two peaks is much shorter than the mean mass-doubling time of wild type fission yeast of 116 minutes [50]. To achieve a longer oscillation period, with increasing cell size, Tyson assumes a dilution of an enzyme that catalyses the breakage of MPF into cdc2 and cyclin-P. Therefore, the orginal model adjusts the rate constant during the cycle so that it is proportional to *exp*(-0.693*t*/*T*_*d*_), where t is the time and *T*_*d *_the doubling time of cell size. In comparison to the amount of intracellular proteins, the size (or the volume) of a cell is a good example for denoting high-level information at cellular level. Hence, implicitly downward causation and the multi-levelness of the system are taken into account by the Tyson model.

We want to make these multiple levels and their interrelation explicit now. Therefore, we introduce an attributed species name *C *that describes the cell and its current volume, i.e. its size. By doing so, we can adjust the rate of MPF dissociation inside the cell dynamically and individually for different cell instances:

Please note, unlike cyclin (*Y *and *Y*_*P*_), we do not distinguish between phosphorylated and unphosphorylated cdc2 (*D*). This is a simplification in accordance with the original model, as the phosphorylation and dephosphorylation reactions of cdc2 are very fast compared to the others and thus can be neglected. Please further note, the constraint (*a *> 1) ensures that *M*_*A *_will never be completely degraded, so that there is at least one molecule of activated MPF at any time. This assumption is needed as otherwise the above rule of MPF activation is not able to fire when the amount of *M*_*A *_is zero. Alternatively, one could introduce an additional cell attribute that denotes the current amount of enclosed *M*_*A *_and serves as high-level information to describe the autocatalytic reaction:

The only processes that are still missing for completing the cell cycle dynamics with varying rates of MPF dissociation, is the growth of a cell, i.e. its volume increase over time, and the abrupt reduction of the volume that mimics cell division. Therefore, we discretize growth in volume to be increased by 1/*T*_*d *_with a rate constant *k*_6 _= 1 per minute, where *T*_*d *_is the mean doubling time:

The cell volume here is only a relative value where 1 and 2 denote typical volumes at birth and division respectively. That is why the above rule for cell growth is constrained to only fire as long as the volume is below 2, i.e. the double value of the typical volume at birth. Consequently, cell division, i.e. halving of the cell's volume, happens after the volume exceeded or is equal to 2:

Please notice that it would be easy to increase the amount of cells here by putting two cells on the right-hand side of the rule. Later we extend the model in this way. However, as the interplay between a high-level state and processes at lower level is subject of our interest here, we keep the cell number constant and just mimic cell division by halving the cell volume. At this stage, the model comprises the intracellular processes shown in [46] and explicit downward causation where the state of a cell (its volume) influcences a process at lower level. However, as Figure 5B depicts, although the mean oscillation period is longer than before and thus closer to observed cell cycle durations, now it is highly variable and still significantly shorter than the mass-doubling time (*T*_*d*_) of 116 minutes. Moreover, this leads to nonconformity of active MPA bursts and cell division times. Hence, to get lifelike oscillatory behavior and to achieve better accordance between protein peaks and division time, we need to further extend the model. Let us therefore take a look on how low-level states influence dynamics at the cell level, i.e. how intracellular dynamics trigger high-level events so that the cell traverses through the different cell cycle phases.

The accumulation of inactive MPF, i.e. a complex where both subunits cyclin and cdc2 are phosphorylated, denotes the initiation of DNA synthesis, i.e. inactive MPF controls the transition from G_1 _to S phase. Therefore, we first equip the cell *C *with an additional attribute for the current phase of the cell cycle to model such transitions. As DNA replication (S phase) takes a rather constant time for each cell cycle and we are more interested in the control of the G_1 _and G_2 _checkpoints, we combine the S and G_2 _phases to a single phase S/G_2_. We then define a rule for the G_1_-to-S/G_2 _transition that is constrained to only fire if the amount of *M*_*I *_exceeds a certain threshold value *t*_7_:

Similarly, the transition from G_2 _to M phase is guarded by a threshold *t*_8 _of the amount of active MPF:

The last transition of the cell cycle changes the phase from M back to G_1_, and in reality, at the same time the cell splits into two daughter cells. However, we are still interested in the interplay between high-level and low-level states only. Thus, we keep the amount of cells constant but reduce the volume of the cell like we've already seen above. The division occurs after active MPF falls below a second threshold value *t*_9 _which is much lower than *t*_8_:

In all three cases of phase transitions, the content of the cell remains the same as the only change governed by the rules is dedicated to the cell cycle phases. The rules describe typical examples of upward causation, i.e. low-level states (the amount of certain protein complexes) determine dynamics at higher levels (the cell). They also show the necessity for flexible reaction constraints to model inter-level causalities.

Now that the model has defined states for the different cell cycle phases, we do not have to restrict the cell to grow in size until its volume has doubled. Instead, we allow the cell to grow at any time but not during the M phase:

Simulation results of this multi-level model show that the mean period of oscillations is now in accordance with the mass-doubling time *T*_*d *_(Figure 5C). Cell division may happen at volumes larger than 2 and consequently the cell cycle may take more time than *T*_*d*_, but if so this has implications for the next cycle. Due to the unusually large volume at birth, MPA activation occurs relatively fast and thus the following cycle tends to be a bit shorter than normal. In this way, upward and downward causation regulates both, the cell cycle duration and cell size homeostasis.

The results emphasize the role of multiple levels and their inter-relation in studying phenomena like cell division. Therefore, ML-Rules provides nesting of species and rates with arbitrary kinetics based on constraints. So far the model appears still to be manageable in other less expressive modeling approaches. However the next step, i.e. moving from a single cell model to multicellular dynamics, illuminates the importance to be able to express multiple levels and their inter-level causalities explicitly and flexibly.

#### Cell division and mating type switching

**Figure 6:**
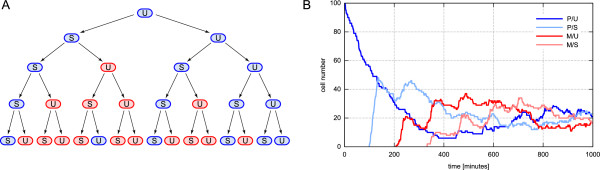
**Mating type switching**. (A) Switching of mating types in a fission yeast cell lineage. Cells of type M are marked with red color, blue stands for mating type P. The unswitchable and switchable states are denoted by U and S respectively. The figure has been redrawn from [58]. (B) Trajectories of a simulation run with an inital population of 100 unswitchable cells of mating type P. Cells are dying with a rate constant *k*_*death *_= 0.006 min^-1^. Mass-doubling time *T*_*d *_= 116 min.

The unicellular fission yeast may undergo sexual reproduction when environmental conditions are getting poor, e.g. when cells are starving. Different mating types (P and M) exist enforcing fusion of cells of opposite types only [51]. The product of fusion is a diploid zygote which rapidly enters a sporulation process. Later, when the environmental conditions improve, spores germinate to spawn haploid cells which then undergo normal asexual proliferation again. The mating type of proliferating cells switches sporadically when a cell divides. This phenomenon is regulated by rather complex mechanisms at gene level [52-57]. However, rather stable phenomenological patterns of switching can be observed [58]. One important characteristic is that cells do not only show one of the two different mating types P or M, but can be also categorized into cells that are able to switch their type and those that are not (Figure 6A).

Although comprising multiple levels, the example model so far describes the dynamics of a single cell only. In order to model a multicellular system, we extend the previous cell division rule (cell cycle transition from M phase to G_1_) to produce two distinct cells. At the same time, instead of modeling detailed processes at genetic level, simple phenomenological alterations of the cell's mating type are assigned according to the regularities depicted in Figure 6A. Therefore, species C is equipped with two additional attributes:

The above rule describes cell division of an unswitchable cell (denoted by the **U **attribute). The complementary schema for division of switchable cells looks pretty much the same and is therefore not shown here. The only differences affect the last attribute of the reactant (**S **instead of **U**) and the assignment for the mating type of the unswitchable product cell: the conditional expression if *t *= **P **then **M **else** P **is assigned which makes the rule valid for matching both mating types P and M. 

Now that we have introduced multiple instances of cells (each with potentially own behavior), it becomes clear that modeling of such systems becomes only viable due to the ability to specify rule schemata. Otherwise one would need to specify defined reaction rules for each potential species, i.e. for each combination of cellular attributes and intracellular protein amounts. This is highly impractical for smaller systems with a finite state space and impossible for systems like the presented one. It shows the importance of rule schemata for supporting multi-level modeling.

The above rule also illustrates the need for binding solutions to variables so that entire solutions can not only be preserved for the reactant species, but can be treated like any other variables and thus also be copied and placed into multiple product species. Unlike the volume of the dividing cell and similar to the single cell division rule in the previous section, the cell's content will not be splitted and distributed among both daughter cells. The contained sub-solution will be entirely copied instead, as (according to the original Tyson model) the total amount of cdc2 protein, i.e. the sum of the amount of species *D*, *M*_*I*_, and *M*_*A*_, is assumed to be constant in each cell. However, by applying specific functions, it would be also possible to split a solution according certain constraints. An integration of such functionality into ML-Rules is planned for the near future.

Mating type switching ensures that - in the long run - both types are equally present in a population of cells. An initial population consisting of only one type of cells nicely shows distinct time points of the first appearance of cells that comprise other combinations of mating type and the ability to switch (Figure 6B). Also the calibration to equally distributed cell types after just a few division cycles can be observed.

#### Pheromone secretion and response

Besides the restriction to cells of opposite mating types, conjugation of fission yeast cells is also regulated by diffusible pheromone molecules [59]. When growing in a nitrogen-poor environment, cells are starving and begin to synthesize mating type specific pheromones that are secreted to the extracellular medium. The pheromone secreted by cells of mating type P is called P-factor and M-type cells produce the M-factor pheromone. Fission yeast cells of different mating type are able to communicate with each other via pheromone molecules. Sensing of pheromones released by the opposite type causes several regulation processes that prepare the cells for mating. One of the main effects is an arrest of the division cycle at the G_1 _phase [60]. We would like to extend our cell model by adding communication via pheromone molecules and the respective responses so that a G_1 _arrest can be observed.

At first, we add some simple rules for pheromone secretion and degradation (diffusion out of the system). For instance, each cell of mating type M produces the M-factor pheromone *F*_*M*_:

M-factor molecules may then influence dynamics of P-type cells in the same solution. Conversely, cells of mating type P may communicate with M-type cells via P-factor molecules (*F*_*P*_). In addition to the M-factor, cells of type M produce and release a P-factor-specific protease (Sxa2) which lowers the effect that P-type cells have on M cells [59,61].

**Figure 7:**
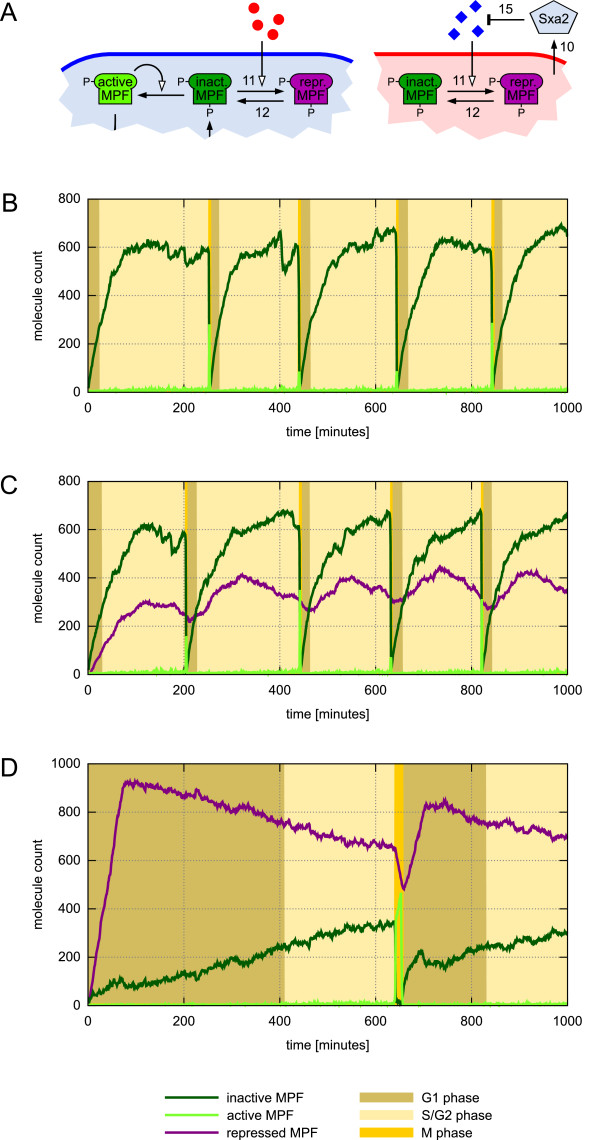
**Pheromone-dependent cell cycle dynamics**. (A) Schematic of MPF repression dependent on the extracellular amount of pheromone molecules. M-factor molecules (red circles) have an effect on cells of mating P, while P-factor (blue diamonds) only influences the intracellular dynamics of M-type cells. P-factor pheromone is catalytically degraded by Sxa2 which is secreted by cells of mating type M only. (B) MPF trajectories and cell cycle phases of a simulation run without pheromones. Mass-doubling time *T*_*d *_= 232 min. (C) An extracellular pheromone amount of 200 molecules reveals no significant difference of MPF and cell cycle dynamics. (D) Large pheromone amount (600 molecules) leads to MPF repression and subsequent adaptation due to increasing cell size. Cell cycle length is significantly increased due to an arrest in the G_1 _phase of the cell cycle.

Instead of modeling a detailed pheromone response signaling cascade including receptor binding, we would like to simply measure the amount of the respective molecules and take this information into account for changing the dynamics of the intracellular control circuit. As already mentioned, pheromones may cause a G_1 _arrest of the cell cycle. It has been shown that inhibition of the cyclin-cdc2 complex is crucial for this process [60]. Therefore, we introduce a new species *M*_*R *_denoting a repressed MPF complex that prevents inactive MPF from being activated (see Figure 7A). The reaction rate of MPF repression is dependent on the amount of extracellular pheromone. The way to describe this is similar to the cell cycle transition rules, where intracellular protein amounts are taken into account in an upward causation manner. However, here we have a downward causation, as MPF resides at a lower level than the pheromones. Also different from the previous examples, the inter-level causation here acts across the boundaries of a nested species, i.e. across the cell membrane, and not just between an attributed species and its enclosed solution:

In fact the above rule includes two different downward causalities at the same time. The first one is the amount of extracellular pheromone, which is included in the rate factor $H=\frac{k_{11}.f^{3}}{k_{11}^{3}+f^{3}}$ describing a Hill type sigmoidal response curve for MPF repression. The second downward causation is a volume-dependence again. This reflects the observation that inhibition of MPF activity is partly lost due to increasing cell size [60], which could, for instance, be a consequence from a dilution of involved (but here not regarded) enzymes.

The single-cell simulation experiments given in Figure 7 show how the added reaction rules influence the intracellular processes and by that have an effect on the dynamics at cell level, i.e. progression through the cell cycle phases. As pheromone secretion and mating takes place when nutrition is poor, the mass-doubling time *T*_*d *_has been increased to 232 minutes. Without pheromone, the cell cyle length then increases to roughly 200 minutes (Figure 7B). Similar dynamics can be observed with a low amount of extracellular pheromone (Figure 7C). The amount of repressed MPF molecules is not enough to have a significant effect on MPF activation. This is different with a higher pheromone concentration. Figure 7D indicates a strong suppression of inactive MPF by the repressed variant. With increasing cell size (not shown), repression gets partly lost, i.e. the cell adapts while it grows and completes the cell cycle finally after more than 600 minutes. Please notice the dramatically increased duration of the G_1_ phase while S/G_2_ and M phase take only slightly more time than without pheromone sensing.

**Figure 8:**
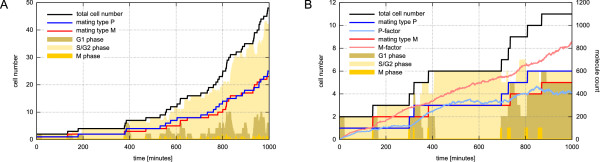
**Cell population growth and pheromone response**. Simulation results of the multicellular model that combines cell division with mating type switching and pheromone response. Mass-doubling time *T*_*d *_= 232 min. (A) Exponential population growth when pheromone production and response is lacking. (B) Pheromone production and response leads to G_1 _arrest and reduced population growth rate.

Compared with exponential population growth unaffected by any pheromones, multicellular simulations with interacting cells show significantly reduced cell numbers, i.e. the mean cell cycle duration is increased (Figure 8). With increasing pheromone concentrations, one can also observe larger fractions of cells being in the G_1_ phase of their cell cycle. After 1000 minutes nearly half the population of cells is arrested in this phase. At this time point, the amount of pheromones is between 400 (P-factor) and 800 (M-factor) molecules. However, although the P-factor-specific protease Sxa2 lowers the amount of P-factor pheromone and thus lowers the effects on M cells, mating type switching ensures equal distributions of cells with mating types P and M.

#### Spatial layer

The model so far assumes all cells as well as each secreted pheromone molecule residing in the same solution, i.e. there is no distinction between different locations. This assumption might be appropriate in many cases. However, especially when it comes to modeling of multicellular systems comprising of communication either via direct cell-to-cell interactions or via diffusible molecules, capturing different species locations might be important [62]. Therefore, to investigate cell division and pheromone signaling in an inhomogeneous solution, we extend the model by some simple spatial dynamics covering pheromone diffusion and different locations of cells.

We adopt the idea of the Next Subvolume Method [63] to add space in a discretized manner. Rules and reaction rates are responsible to describe reactions between molecules and their diffusion into another voxel in the spatial grid. A new attributed species *G *is introduced, which represents virtual reaction compartments within a two-dimensional grid. Each voxel *G *may comprise a solution of cells and pheromone molecules with a homogeneous distribution like before, but species may migrate to adjacent voxels according to certain rules (see Figure 4 for a schematic description of the spatial setting). 

The initial solution comprises *x*_*max *_× *y*_*max *_(with *x*_*max*_*, y*_*max *_∈ ℕ) species *G*, each with a unique combination of attribute values *G*(*x, y*) with *x *∈ {1,..., *x*_*max*_} and *y *∈ {1,..., *y*_*max*_}. In this way, a defined relationship between each species *G *is specified to represent the spatial coordinates of a two-dimensional grid. Diffusion of molecules can then be described by simply moving the species from one voxel to an adjacent one. A constraint comparing the coordinates of two species *G *guarantees that migration takes place between neighbored locations only. The rule schema for diffusion of P-factor pheromone in a von Neumann neighborhood looks as follows:

with *nb*(*x*_1_*, y*_1_*, x*_2_*, y*_2_) *= *if (*x*_1 _*= x*_2 _∧ (*y*_1 _= *y*_2 _+ 1 ∨ y_1 _= *y*_2 _- 1)) ∨ (*y*_1 _*= y*_2 _∧ (*x*_1 _= *x*_2 _+ 1 ∨ *x*_1 _*= x*_2 _- 1)) then **true **else **false**.

Like before, the model shall still include diffusion out of the system, i.e. for particle diffusion the grid shall be an open system. Therefore, another rule checks whether a certain voxel is part of the boundary of the grid. If so, a pheromone molecule is simply removed with a certain probability:

Besides pheromone diffusion, we would like to also describe different locations of cells. However, instead of random diffusion we would like to model some sort of *excluded volume effect *to avoid that too many cells occupy a voxel. Therefore, if a location gets crowded, cells may be pushed to an adjacent less crowded voxel. In principle, the rule for such a displacement from crowded areas looks quite similar to rules that describe diffusion. Constraints guarantee moving under certain conditions only, e.g. to a neighboring voxel only, and the kinetic rate depends on the amount of species, i.e. cells. The main difference is that due to the fact that cells typically have different attributes and sub-solutions, we can not use a species identifier to get the total number of cells within a solution. Therefore, an additional attribute of *G *is introduced that holds the current number of cells in each voxel:

The number of cells is a high-level property of *G *and can be used to specify the probability with which a cell may move to an adjacent location. The rate of the above rule makes migrations to empty locations more likely than those to crowded ones. Please notice the assignments of values (*n*_1 _- 1 and *n*_2 _+ 1) for updating the current cell number of each voxel when the rule fires. As the number of cells may also change due to cell division and death, the according rules have to be extended such that they have an extended context where the attribute of *G *can be explicitly manipulated. For example, the cell division rule for an unswitchable cell looks as follows:

An elegant alternative to the above strategy would be the already discussed concept of applying functions to solutions, so that the amount of cells in a given solution can simply be counted. In any case, the examples show how spatial effects like diffusion and excluded volume can be modeled in an ad hoc way, although ML-Rules has been developed without explicit notions of space. Approaches which are aimed at spatial rule-based modeling explicitly deal with such problems and emphasize the need for describing spatial phenomena for larger entities [64,65].

Simulation of population growth within the described spatial setting reveals that the overall ratio between the different mating types remains nearly constant over time. However, local differences in the amount of P and M type cells can be observed (see additional file 2).

### Related work

The aim of this paper has been to identify essential concepts for rule-based multi-level modeling, to present their realization in ML-Rules, and to show their role based on a case study. ML-Rules could built on ideas developed in a rule-based approach for multi-level modeling of ecological systems [66] where attributes, components of specific types, and interfaces are assigned to individual entities. The approach combines hierarchical nesting and the description of constrained dynamics in terms of rule schemata. ML-Rules shares also central features with the rule-based formalism React(C), which supports molecules with attribute values of any type and reaction constraints to flexibly define reaction rates [15]. Being of arbitrary type, attributes can also encode solutions and thus hierarchically nested entities. However, React(C) has no notion of nesting: rules cannot be applied to a solution *nested within *an entity. Instead, rules can only be applied to an entity *attributed with *a specific solution, i.e. top-down. Considering that nested hierarchies may be dynamically changed, e.g. in models describing vesicles that fuse with membranes, this limits the expressive power of React(C) with respect to multi-level modeling. 

Recently, Oury and Plotkin have presented a stochastic multi-level multiset rewriting language [67], in which rules can be applied to nested species to support multi-level modeling in systems biology. However, downward and upward causation cannot easily be expressed, because attributes and corresponding constraints on reactions are not yet supported. However, this is announced among the next steps to do. 

As has been already discussed, ML-Rules does not provide an explicit notion of linkage, e.g. to describe bonds within protein complexes. Hierarchical graphs with multiple edge types offer a natural and explicit representation of such bindings within hierarchical model structures. In this context, a generalized graph isomorphism and labeling algorithm like HNauty [68] may be of particular importance. Although originally developed for the structured annotation of flat rule-based models, hierarchical graphs and the HNauty algorithm are promising techniques for the development of an efficient multi-level approach based on graph-rewriting rules.

Spatial structuring of models shares general concepts with multi-level modeling as different levels are defined by a separation from each other in a broader sense. In systems biology, the most common representation of space is realized by simple compartmentalization, i.e. by separating different chemical solutions from each other and allowing for basic transport rules to change the location of molecules, see e.g. BIOCHAM [69] and little b [70]. More sophisticated capabilities for membrane-mediated transport and interaction rules are supported by cBNGL [71], an extension of the original BioNetGen language [11]. Structures and rules in cBNGL are tightly coupled with the concept of compartments and membranes, e.g. the language distinguishes between three-dimensional (compartment volume) and two-dimensional (surface, i.e. membrane) compartments.

Other approaches focus on supporting dynamic compartment structures. BioAmbients [72], for example, which is based on the π-calculus [26], supports wrapping of processes by so called ambients. Both, processes and ambients, are allowed to enter or exit other ambients and two ambients are allowed to merge into a single one. Similarly, the bio*κ*-calculus [73] also allows to fuse multiple membranes resulting in a single compartment. It aims at combining rule-based modeling with dynamic membrane formalisms like the Brane Calculi [74] and P systems [75]. However, although rooted in the rule-based domain, bio*κ *shows limited expressiveness for modeling biochemical systems compared to other rule-based languages, e.g. the *κ*-calculus [7]. Another rule-based formalism with explicit means for dynamic nested model structures is the Calculus of Wrapped Compartments [76,77]. None of these approaches equips compartments with a state and a behavior of their own; dynamics at the level of compartments are initiated by the enclosed processes or rules in a "bottom up" way.

Bigraphs [78] is different from the above formalisms as there is no distinction between structural and behavioral elements of a model and thus the approach pursued is rather similar to ML-Rules. Each node of a bigraph may be enclosed by another node and may contain further nodes itself. So, nesting is an inherent property of the bigraphical components. Equipped with a stochastic semantics [79], reactive bigraphs have been successfully applied for modeling cell biological systems. However, the state of a node is defined by its linkage to other nodes only. Also the lack of constraints on reactions limits the modeling of inter-level causalities.

Beyond ordinary compartment-like spatial approaches (to which we would also count ML-Rules, although its expressiveness widens the applicability for describing other spatial relationships as well), diverse methods have been developed for modeling and simulation of more complex spatial phenomena. Smoldyn, for example, supports simulation of spatial compartments with diffusing molecules, membrane interactions, and excluded volume effects [80]. The latter is also an important feature of ML-Space, a modeling and simulation approach that supports hierarchical nesting and combines population-based reaction-diffusion systems with individual particles for representing different spatial resolutions [65]. Meredys is another simulator that supports reaction-diffusion events taking place in multiple compartments. However, the main feature of Meredys is that molecules and molecular complexes may have realistic shapes in two and three dimensions [81].

Multi-level models that comprise a wide range of spatial scales, e.g. from the molecular to tissue or even organ scale, often need to consider different spatial relationships at different levels. For example, while at the molecular level well-stirred compartments or heterogenously distributed reaction-diffusion systems are appropriate representations, modeling the dynamics at tissue level might need to take physical mechanics of interacting cells into account. The strong diversity in applied methods is one reason why such multi-scale models typically lack a unifying formal modeling language. Instead, different model parts are described and interpreted differently. To integrate these different parts efficiently, either monolithic mixtures of model descriptions and simulators are programmed from scratch or specialized multi-scale software platforms are used that have been developed for certain applications, see e.g. [82-86]. 

MGS provides integration of explicit descriptions of space in a generic uniform setting [87,88]. The approach combines rule-based modeling with topological collections to specify which and how model entities may interact with each other. Various topological collections define different local relationships of individual entities, e.g. ordinary multisets or Delaunay triangulation. Although MGS does not have an inherent notion of nesting, its underlying concepts allow to describe multi-level models in a versatile manner and across various spatial scales.

The need to describe systems at different levels has also been addressed by Petri nets approaches, see e.g. [89-91]. For instance, HORNETS (Higher Order Reference Nets) is a formalism that allows to have Petri nets as tokens of Petri nets [91]. However, HORNETS are executed in equidistant time steps as they are aimed at modeling software systems, e.g. workflows, rather than biochemical systems.

## Conclusions

Rule-based languages are a suitable starting point for developing a concise and compact language for multi-level modeling of cell biological systems. Therefore, a combination of concepts, part of which are already well established, can be exploited.

Rule schemata help reducing the size of models and equally important, add the required flexibility to express dynamics at different levels in a general manner. Nesting species, assigning attributes to these species, and constraining reactions according to attributes have been identified as further essential ingredients in supporting multi-level modeling. Species are described by attributes and the species they contain. Both of which might constrain rules (due to functions and conditional expressions) or be altered by them. Thereby, the boundaries of levels might be crossed.

How dynamics at different levels can be described in a rule-based approach has been shown with a model of fission yeast to analyze the regulations between cell cycle control, cell division, mating type switching, and cell-cell communication via diffusible pheromone molecules.

The concepts have been realized in ML-Rules which has been implemented in JAMES II. The use of a plug-in-based modeling and simulation framework has allowed a rapid prototyping of a suitable modeling and simulation environment for our experiments. However, the current simulator is a prototype and realizes a purely stochastic discrete event approach. Although JAMES II offers a coarse grained parallel execution - e.g. to speed up multiple simulation runs - already the single run execution of a more complex ML-Rules model, like the presented multicellular fission yeast model, takes a significant amount of time in the current implementation. This is due to the expressiveness of ML-Rules which requires specific effort to keep calculation costs at bay. Thus, the next steps with respect to implementation will be to look into exploiting different variants of the SSA algorithm, e.g. the optimized direct method, speeding up the matching of reactants, and exploring the potentials of an alternative Monte Carlo method as well as hybrid approaches. 

From the modeling point of view, the developed concepts shall be put to test in concrete applications that might be difficult to describe with currently available modeling approaches. For example, potential application areas for ML-Rules are various systems where the relation between intracellular and intercellular dynamics play a role, e.g. quorum sensing, tumor growth, and plant root growth. The presented approach appears also suitable for modeling dynamic processes with multiple membrane bound compartments, like endocytosis, active vesicle transport along cytoskeletal filaments, and processes at the Golgi apparatus.

## Authors' contributions

Ideas and concepts were jointly discussed among all authors. The manuscript was also jointly written. CM identified central requirements for multi-level modeling in cell biological systems, developed the model, and executed the corresponding simulation study. SR implemented the model editor and simulator for ML-Rules in JAMES II. All authors read and approved the final manuscript.

## Supplementary Material

Additional file 1 **ML-Rules demo program **The ZIP file comprises a prototype tool of ML-Rules including a model editor, the simulator, and a rudimentary line chart visualization of simulation trajectories. Also a user manual and several example models are part of the tool package. To start the demo tool, please unzip the file and execute the run.jar file. Java Runtime Environment (Version 6 or higher) is required for execution. Click here for file

Additional file 2 **Example models **The PDF file contains descriptions of the entire example models including initial solutions and parameter values that have been used for the simulation studies. Click here for file
