# Supplementary material for: Rule-based multi-level modeling of cell biological systems
Source: BMC Syst Biol. 2011 Oct 17;5:166. doi: 10.1186/1752-0509-5-166 (PMC3306009; doi:10.1186/1752-0509-5-166)
Supplement: Additional file 1 — ML-Rules demo program The ZIP file comprises a prototype tool of ML-Rules including a model editor, the simulator, and a rudimentary line chart visualization of simulation trajectories. Also a user manual and several example models are part of the tool package. To start the demo tool, please unzip the file and execute the run.jar file. Java Runtime Environment (Version 6 or higher) is required for execution. [file 1752-0509-5-166-S1.ZIP › ML-Rules/manual.pdf]

ML-Rules demo tool – user manual

This manual is intended to help users working with the ML-Rules demo tool. Besides explaining the concrete syntax, many simple examples illustrate how to use ML-Rules for modeling diverse biochemical and multi-level systems. However, we would like to refer to the main paper for a comprehensive description of the main ideas and methods behind ML-Rules. The demo tool provides a concrete implementation of ML-Rules including a text-based model editor, simulator, a simple line chart visualization, and simulation data output both as in a CSV and XML file format. It does not provide full functionality of the JAMES II modeling and simulation framework on which it is based on. The source code with full integration of ML-Rules into JAMES II, including different simulator implementations and data output options, sophisticated experiment set-ups, parameter estimation routines and support for model validation, is planned to be released soon on `http://www.jamesii.org`.

Contents

1 Getting started **2**

1.1 System requirements 2
1.2 Running the tool 2
1.3 The main window 2

2 Elements of a model description **3**

2.1 Parameters 3
2.2 Species definitions 3
2.3 Initial solution 3
2.4 Rule schemata 4

3 Non-hierarchical systems and rules (basics) **4**

3.1 Simple (biochemical) reactions 4
3.2 Alternative kinetics 5
3.3 Reaction constraints 5
3.4 Complexation via unique bonds 5

4 Hierarchical model structures **6**

4.1 Nested solutions 6
4.2 Preserving (rest) solutions 6
4.3 Migration 6

5 Simulation and data output **7**

1 Getting started

1.1 System requirements

The ML-Rules demo tool is written in Java (compiled to Java bytecode) and should therefore run on every major operating system (incl. Microsoft Windows, Apple MacOS X, and Linux) for which the Java Runtime Environment (Version 6 or higher) is available.

1.2 Running the tool

If not yet done, first unzip the `ML-Rules.zip` archive to a directory of your choice. To run the tool, in most cases it is sufficient to double-click the `run.jar` file. If this does not work, type in and enter the following command into a terminal console:

```
java -jar run.jar
```

1.3 The main window

After starting the tool, the main window including the model editor appears (Figure 1). Model description files can be opened and saved. The “examples” directory contains several (simple and more complex) example models. The textual editor includes syntax highlighting and on-the-fly checking for warning the user if something is syntactically wrong. Also obvious semantical inconsistencies will generate according messages. At the bottom of the main window, buttons for starting and stopping simulation runs exist.

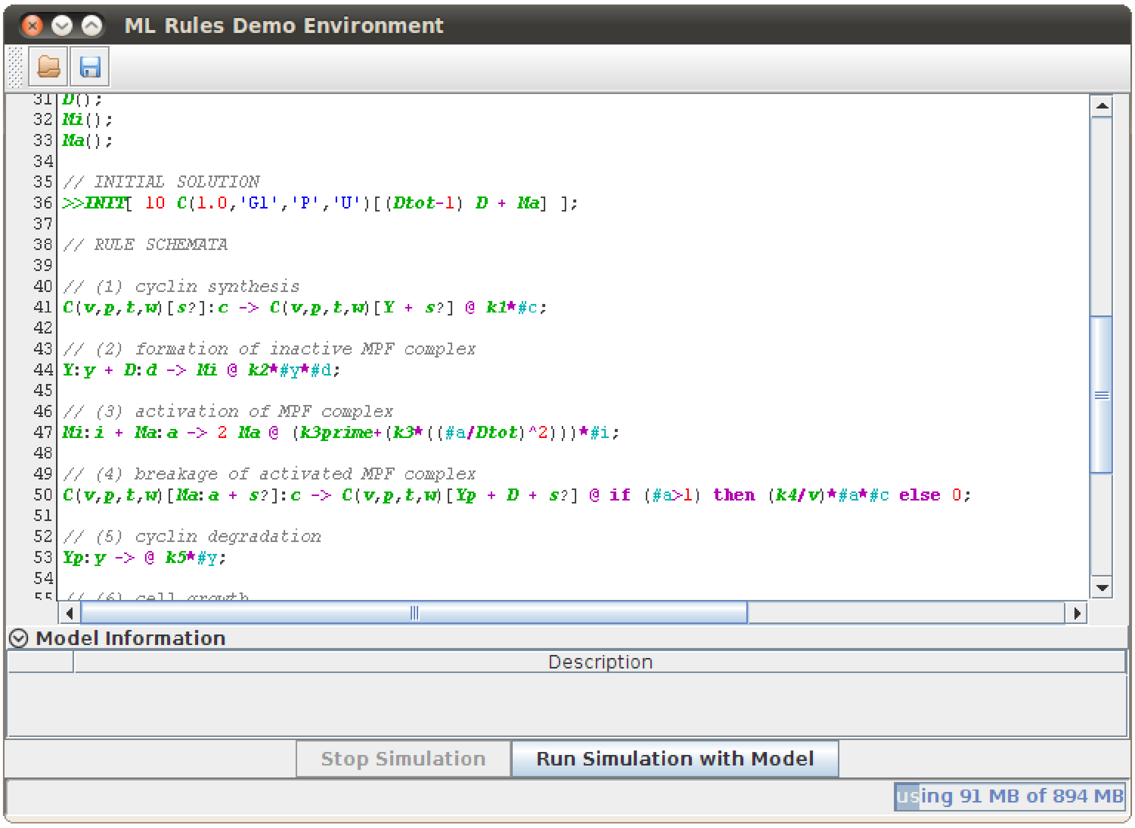

Figure 1: Main window of the ML-Rules demo tool.

2 Elements of a model description

A model description consists of four different kinds of elements that have to be described in the following order:

1. Parameters (optional)
2. Species definitions
3. Initial solution
4. Rule schemata

At any point in the model description, comments can be inserted by using the double-slash command, for example:

```
// this is a comment
```

2.1 Parameters

At the beginning of a model description a list of optional constant model parameters can be specified. Each parameter definition consists of a name followed by a colon and its value. Finally, a semicolon marks the end of the parameter definition. A parameter can be referenced from any element in the model description and – like attributes of species – can be a numerical value or a string (letters and/or digits embraced by ''). Expressions are also allowed to define parameters. Here are some valid examples:

```
k1:1.0;
k2:2.63*1e-4;
NCells:5000;
NVirus:NCells/100; //the number of viruses is 50
stateX:'meth1';
```

2.2 Species definitions

Species definitions specify the type of model entities that are defined by their name and their arity, i.e. the number of attributes. The arity of each species is defined by a non-negative integer value within a pair of parentheses following its name. For example, a species `A` with one attribute and a species `B` without attributes are specified as follows:

```
A(1);
B(0);
```

2.3 Initial solution

The initial model state is defined by the initial solution `>>INIT[...]`. Distinct species are separated by a `+` symbol and the amount of a species can be specified by an integer number put in front. The following example specifies an inital solution comprising two species of type `A` (one with attribute `'u'` and one with `'p'`), each of them with an amount of 1000, and the third species is the non-attributed `B` with an initial amount of 200.

```
>>INIT[
  1000 A('u') +
  1000 A('p') +
  200 B
];
```

2.4 Rule schemata

Rules (or rule schemata) define the dynamics of a model. A rule (schemata) consists of a multiset of reactants, a multiset of products, and a stochastic rate. The first two parts are separated by `->` and the rate follows the `@` symbol:

```
reactants -> products @ rate;
```

Concrete examples will be given below.

3 Non-hierarchical systems and rules (basics)

3.1 Simple (biochemical) reactions

A simple biochemical reaction typically follows the law of mass action where the amount of reactant species determines the speed of the reaction, i.e. the rate of the reaction rule. By assigning an identifier to each reactant, its amount can be accessed for specifying the rate (but also for defining the products, e.g. by assigning certain attributes). The special variable `#i` holds the current amount of a matched species that has assigned an identifier `i`. For example, the following rule describes a degradation reaction of `B` with a reaction rate constant `k`:

```
B:b -> @ k * #b;
```

Please note, parentheses behind the species name are not necessary if the number of attributes is zero. Degradation rules for an attributed species look as follows:

```
A('u'):a -> @ k * #a;
A('p'):a -> @ k * #a;
```

Instead of specifying each potential state of `A` explicitly, one can specify a schematic rule with a variable rather than a defined attribute value:

```
A(x):a -> @ k * #a;
```

Applied to a solution that comprises both species `A('u')` and `A('p')` would then lead to two rule instantiations that are equivalent to the two explicit rules above.

Missing in the above degradation examples, the state of a species can be modified by simply putting a product species to the right-hand side of the rule. An auto-phosphorylation reaction of species `A` may look as follows:

```
A('u'):a -> A('p') @ k * #a;
```

The previous rules describe first order reactions only, i.e. reactions that depend on the amount of a single species. Bimolecular reactions can be specified in a similar way. The only difference is, that multiple reactants (equally to multiple products) are separated by a `+`:

```
1 A('p'):a + B:b -> ApB @ k * #a * #b;
2 A('u'):a1 + A('p'):a2 -> A('p') + A('p') @ k * #a1 * #a2;
```

The latter reaction rule in row 2 can be also specified in a more compact manner by using a stoichiometric factor for describing the products:

```
A('u'):a1 + A('p'):a2 -> 2 A('p') @ k * #a1 * #a2;
```

Stoichiometric factors can be also used to describe unimolecular reactions with identical reactant species:

```
2 B:b -> BB @ k * binom(#b,2);
```

Please note the binomial coefficient function to correctly describe the mass action kinetics of such an unimolecular reaction.

3.2 Alternative kinetics

The previous example reactions all follow the law of mass action. However, ML-Rules allows to specify arbitrary stochastic rates in a flexible manner so that alternative rate kinetics like Michaelis-Menten or Hill-type kinetics can be easily described. For example, an enzymatic reaction

$$E + S \underset{k_2}{\overset{k_1}{\rightleftharpoons}} ES \xrightarrow{k_3} E + P$$

can be either described by three detailed mass action rules or in an approximated way by assuming a quasi-steady state of the very fast binding/unbinding events between enzyme `E` and substrate `S`:

```
// mass action kinetics
E:e + S:s -> ES    @ k1 * #e * #s;
ES:c      -> E + S @ k2 * #c;
ES:c      -> E + P @ k3 * #c;

// Michaelis-Menten kinetics
E:e + S:s -> E + P @ (k3 * #e * #s) / (KM + #s);
```

3.3 Reaction constraints

Besides specifying prerequisites for firing by defined attributes, rules can be further constrained in a flexible manner. For example, one can use the `if-then-else` conditional expression for constraining a rule to only fire if a certain threshold amount `T` of a reactant species `A('p')` is exceeded:

```
A('p'):a -> @ if (#a>T) then k*#a else 0;
```

Nested conditions are also possible just as conditional constraints to specify attributes of reactants and products. For example, the following rule describes switching between the two states of `A`, i.e. the assigned attribute of the product depends on the attribute of the matched reactant species:

```
A(x):a -> A(if (x=='p') then 'u' else 'p') @ k*#a;
```

3.4 Complexation via unique bonds

Links between molecules or other entities can be used to reduce model complexity and to preserve states of attributed species when modeling binding reactions. Therefore, by using the `$` command, a fingerprint-like unique value can be created and assigned to each binding partner:

```
A(x,0):a + B(0):b -> A(x,$link) + B($link) @ k_bind * #a * #b;
```

Please notice, both species need additional binding site attributes and the value `0` is assumed to indicate the unbound state in this example. For the backward reaction (dissociation) one only needs to match reactants that share the same unique value (and is not `0`, i.e. unbound):

```
A(x,y) + B(y) -> A(x,0) + B(0) @ if (y!=0) then k_unbind else 0;
```

A multiplication of the rate constant `k_unbind` with the amount of the reactant species is not needed, as due to the assignment of a newly created unique value for each complex, their amount will be 1 in any case.

Please note that the simulation performance may be slowed down by modeling complexes with unique links as the number of species may increase dramatically and therefore the time needed for matching reactants may also increase. Creation and assingment of unique values can be also used to individualize certain species, e.g. to observe state changes of an individual entity over time.

4 Hierarchical model structures

4.1 Nested solutions

The main difference between flat and hierarchical models is the existence of sub-solutions contained by at least some of the species. Therfore, square brackets indicate that a species encloses further species. For example, a nested species Cell that encloses a Nucleus looks as follows:

```
Cell[Nucleus]
```

Accordingly, specification of a nested initial model state consisting of 2000 molecules A and 200 molecules B, both enclosed by the Cell and the Nucleus species, is straightforward:

```
>>INIT[
  Cell[2000 A + 200 B + Nucleus[2000 A + 200 B]]
];
```

Of course, species in a hierarchical model can still have attributes. If nested species have attributes, the square brackets follow the round ones:

```
>>INIT[
  Cell(1.0)[1000 A('u') + 1000 A('p') + 200 B +
    Nucleus[1000 A('u') + 1000 A('p') + 200 B]
  ]
];
```

Although rule schemata for multi-level models are basically similar to rules that can be found in flat models, there are also some differences that are explained below.

4.2 Preserving (rest) solutions

When applying rules to nested species, i.e. species which contain a sub-solution, one typically might want to preserve the entire sub-solution without specifying its defined content. Therefore, a variable with the suffix ? can be specified and re-inserted into the product. For example, the following rule describes an abstract process of cell growth (increasing an attribute value of a Cell species) by which the whole content of the cell remains untouched:

```
Cell(v)[sol?]:c -> Cell(v+1)[sol?] @ k_growth * #c;
```

Similarly, when certain species of a sub-solution lie in the focus of a rule schema, one might want to preserve the rest of the solution, i.e. the whole sub-solution minus explicitly defined reactant species:

```
Nucleus[B:b + sol?]:n -> Nucleus[sol?] @ k_degrad_BNuc * #b;
```

4.3 Migration

Rules can be defined for describing migration of species, i.e. entry into or exit from a nested species. For example, a particle P enters a Cell by crossing the membrane:

```
P:p + Cell[sol?]:c -> Cell[P + sol?] @ k_enter * #p * #c;
```

Endocytosis can be modeled by creation of an Endosome that encloses the entering particle P:

```
P:p + Cell[sol?]:c -> Cell[Endosome[P] + sol?] @ k_endo * #p * #c;
```

Also, entire solutions may migrate. For example, during exocytosis, a Vesicle fuses with the cell membrane and thereby releases its content to the extracellular solution:

```
Cell[Vesicle[sV?]:v + sC?]:c -> Cell[sC?] + sV? @ k_exo * #v * #c;
```

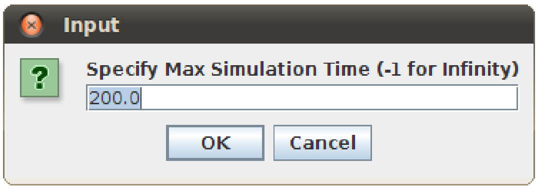

Figure 2: Simulation time specification.

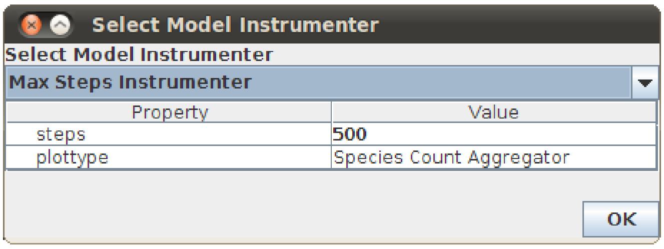

Figure 3: Instrumenter selection.

5 Simulation and data output

To simulate a model, press the button "Run Simulation with Model" at the bottom of the main window (see Figure 1). A new window appears where you can specify the maximal simulation run time (Figure 2). Press "OK" to proceed.

Now you have to select a model instrumenter that specifies how simulation data shall be observed (Figure 3). A careful instrumentation allows to reduce the overhead for observing data (resulting in a faster simulation) as well as the amount of data (to save disc space, for example), while keeping the resolution of data as accurate as needed. Choose one of the following three instrumenters:

- **Max Steps Instrumenter:**
  Takes the maximal simulation run time and its "steps" property into account to calculate the time interval at which data shall be observed. For example, 2000 steps and a maximal simulation time of 200 generates trajectories with a resolution of 0.1 time units.

- **Time Step Instrumenter:**
  The resolution of model observation can be specified by giving a defined time interval.

- **Each Step Instrumenter:**
  Model observation at each step, i.e. with highest resolution. Caution: depending on the model, this may slow down simulation runs significantly and exported data files may get very large.

With the "plottype" property the type of data aggregation for an on-the-fly visualization can be specified. Choose one of the following plot types:

- **Species Count Aggregator:**
  Aggregates all species with the same name, not matter which attributes they have and at which level and within which solution they reside.

- **Species Attribute Aware Count Aggregator:**
  Aggregates all species with identical names and the same combination of attributes. A distinction between different levels or solutions is not possible.

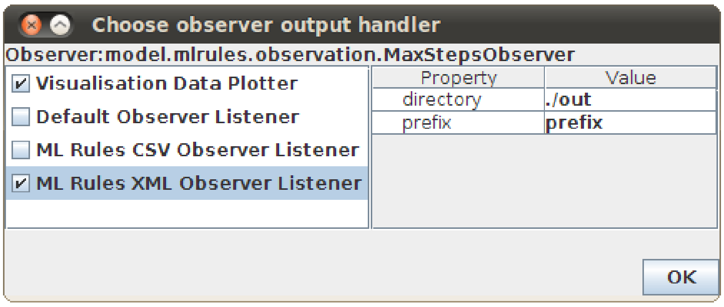

Figure 4: Observer selection.

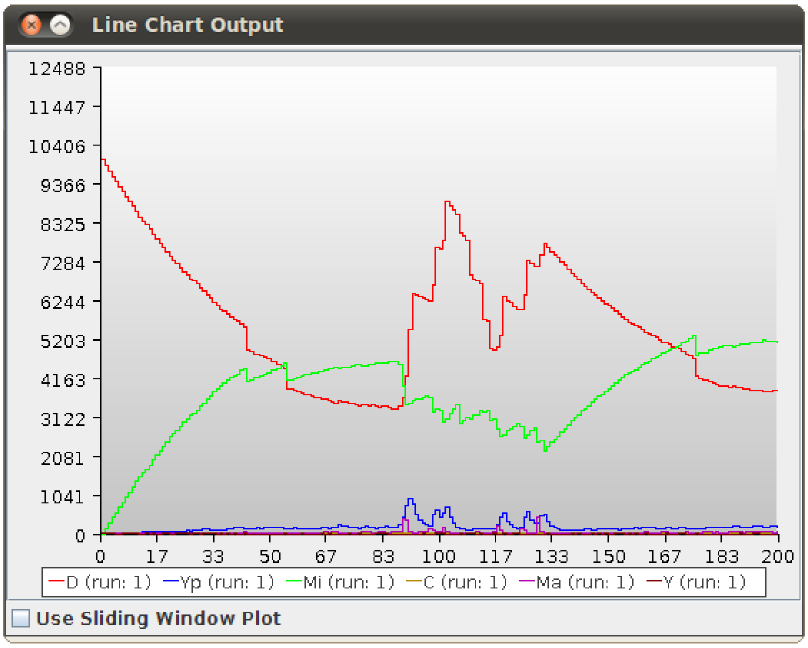

Figure 5: Line chart output.

- **Species Hierarchy Count Aggregator:**
  Aggregation by species names residing at the same level. Different solutions at the same hierarchical level will not be taken into account.
- **Species Hierarchy Attribute Aware Count Aggregator:**
  Aggregates all species with identical names and the same combination of attributes residing at the same hierarchical level. No distinction between different solutions at the same level.

Finally, observer output handlers can be chosen (Figure 4). "Visualisation Data Plotter" enables on-the-fly visualization of the simulation run by opening an extra window (see Figure 5). Both, the "ML Rules CSV Observer Listener" and the "ML Rules XML Observer Listener", export simulation data to CSV and XML files respectively. The XML file format encodes the entire model state including species attributes and the model hierarchy. An output directory and file name prefix can be specified.
