# Supplementary material for: Rule-based multi-level modeling of cell biological systems
Source: BMC Syst Biol. 2011 Oct 17;5:166. doi: 10.1186/1752-0509-5-166 (PMC3306009; doi:10.1186/1752-0509-5-166)
Supplement: Additional file 2 — Example models The PDF file contains descriptions of the entire example models including initial solutions and parameter values that have been used for the simulation studies. [file 1752-0509-5-166-S2.PDF]

Multi-level model of fission yeast cell cycle regulation, mating type switching, and pheromone response

The following abstract rule specifications, initial solutions, and parameter settings present whole model descriptions of the fragmentious examples in the paper. They have been used for performing simulation experiments of which some results are depicted in Figures 5-8 in the paper.

1 Single-cell model of fission yeast cell cycle regulation (Figure 5C)

1.1 Species names

- fission yeast cell: C with $ar(C) = 2$
- unphosphorylated cyclin: Y with $ar(Y) = 0$
- phosphorylated cyclin: Y_P with $ar(Y_P) = 0$
- cdc2: D with $ar(D) = 0$
- inactive MPF: M_I with $ar(M_I) = 0$
- active MPF: M_A with $ar(M_A) = 0$

1.2 Rule schemata

Cyclin synthesis and formation of inactive MPF complex (1-2)

- $C(v,p)[s]^c \xrightarrow{k_1 \cdot c} C(v,p)[Y + s]$
- $Y^y + D^d \xrightarrow{k_2 \cdot y \cdot d} M_I$

MPF activation (3)

- $M_I^i + M_A^a \xrightarrow{(k_3' + k_3 (a/D_{tot})^2) i} 2\, M_A$

Breakage of activated MPF complex and cyclin degradation (4-5)

- $C(v,p)[M_A^a + s]^c \xrightarrow[a>1]{(k_4/v) a \cdot c} C(v,p)[Y_P + D + s]$
- $Y_P^y \xrightarrow{k_5 \cdot y} \emptyset$

Cell growth (6)

- $C(v,p)[s]^c \xrightarrow[p \in \{\mathbf{G1}, \mathbf{SG2}\}]{k_6 \cdot c} C(v + (1/T_d), p)[s]$

Cell cycle transitions (7-8)

- $C(v, \mathbf{G1})[M_I^i + s]^c \xrightarrow[i>t_7]{k_7 \cdot c} C(v, \mathbf{SG2})[M_I + s]$
- $C(v, \mathbf{SG2})[M_A^a + s]^c \xrightarrow[a>t_8]{k_8 \cdot c} C(v, \mathbf{M})[M_A + s]$

Cell division (9)

- $C(v, \mathbf{M})[M_A^a + s]^c \xrightarrow[a<t_9]{k_9 \cdot c} C(\frac{v}{2}, \mathbf{G1})[M_A + s]$

1.3 Initial solution

$C(1.0, \mathbf{G1})[D_{tot} - 1)\, D + M_A]$

1.4 Parameters

Parameter	Value	Description
D_{tot}	1000 molecules	total amount of cdc2 molecules
T_d	116 min	mass-doubling time
k_1	$0.015 \cdot D_{tot}$ min^{-1}	rate constant for cyclin synthesis
k_2	200 min^{-1}	rate constant for inactive MPF formation
k_3'	0.018 min^{-1}	rate constant for basal MPF activation
k_3	180 min^{-1}	rate constant for MPF activation
k_4	4.5 min^{-1}	rate constant for MPF dissociation
k_5	0.6 min^{-1}	rate constant for degradation of phosphorylated cyclin
k_6	1.0 min^{-1}	rate constant for cell growth
k_7	10^6 min^{-1}	rate constant for G_1-to-S/G_2 transition
k_8	10^6 min^{-1}	rate constant for S/G_2-to-M transition
k_9	10^6 min^{-1}	rate constant for M-to-G_1 transition
t_7	250 molecules	threshold for G_1-to-S/G_2 transition
t_8	70 molecules	threshold for S/G_2-to-M transition
t_9	20 molecules	threshold for M-to-G_1 transition

2 Multi-cellular model of cell cycle regulation, cell division, and mating type switching (Figure 6B)

2.1 Species names

- fission yeast cell: C with $ar(C) = 4$
- unphosphorylated cyclin: Y with $ar(Y) = 0$
- phosphorylated cyclin: Y_P with $ar(Y_P) = 0$
- cdc2: D with $ar(D) = 0$
- inactive MPF: M_I with $ar(M_I) = 0$
- active MPF: M_A with $ar(M_A) = 0$

2.2 Rule schemata

Cyclin synthesis and formation of inactive MPF complex (1-2)

- $C(v,p,t,w)[s]^c \xrightarrow{k_1 \cdot c} C(v,p,t,w)[Y+s]$
- $Y^y + D^d \xrightarrow{k_2 \cdot y \cdot d} M_I$

MPF activation (3)

- $M_I^i + M_A^a \xrightarrow{(k_3' + k_3 (a/D_{tot})^2) i} 2\, M_A$

Breakage of activated MPF complex and cyclin degradation (4-5)

- $C(v,p,t,w)[M_A^a + s]^c \xrightarrow[a>1]{(k_4/v) a \cdot c} C(v,p,t,w)[Y_P + D + s]$
- $Y_P^y \xrightarrow{k_5 \cdot y} \emptyset$

Cell growth (6)

- $C(v,p,t,w)[s]^c \xrightarrow[p \in \{\mathbf{G1}, \mathbf{SG2}\}]{k_6 \cdot c} C(v + (1/T_d), p, t, w)[s]$

Cell cycle transitions (7-8)

- $C(v,\mathbf{G1},t,w)[M_I^i + s]^c \xrightarrow[i>t_7]{k_7 \cdot c} C(v,\mathbf{SG2},t,w)[M_I + s]$
- $C(v,\mathbf{SG2},t,w)[M_A^a + s]^c \xrightarrow[a>t_8]{k_8 \cdot c} C(v,\mathbf{M},t,w)[M_A + s]$

Cell division (9)

- $C(v,\mathbf{M},t,\mathbf{U})[M_A^a + s]^c \xrightarrow[a<t_9]{k_9 \cdot c}$
  $C(\frac{v}{2},\mathbf{G1},t,\mathbf{S})[M_A + s] + C(\frac{v}{2},\mathbf{G1},t,\mathbf{U})[M_A + s]$
- $C(v,\mathbf{M},t,\mathbf{S})[M_A^a + s]^c \xrightarrow[a<t_9]{k_9 \cdot c}$
  $C(\frac{v}{2},\mathbf{G1},t,\mathbf{S})[M_A + s] + C(\frac{v}{2},\mathbf{G1},\texttt{if}\ t = \mathbf{P}\ \texttt{then}\ \mathbf{M}\ \texttt{else}\ \mathbf{P},\mathbf{U})[M_A + s]$

Cell death

- $C(v,p,t,w)^c \xrightarrow{k_{death}\cdot c} \emptyset$

2.3 Initial solution

$init_C\, C(1.0, \mathbf{G1}, \mathbf{P}, \mathbf{U})[(D_{tot}-1)\, D + M_A]$

2.4 Parameters

Parameter	Value	Description
D_{tot}	1000 molecules	total amount of cdc2 molecules
T_d	116 min	mass-doubling time
k_1	$0.015 \cdot D_{tot}$ min^{-1}	rate constant for cyclin synthesis
k_2	200 min^{-1}	rate constant for inactive MPF formation
k_3'	0.018 min^{-1}	rate constant for basal MPF activation
k_3	180 min^{-1}	rate constant for MPF activation
k_4	4.5 min^{-1}	rate constant for MPF dissociation
k_5	0.6 min^{-1}	rate constant for degradation of phosphorylated cyclin
k_6	1.0 min^{-1}	rate constant for cell growth
k_7	10^6 min^{-1}	rate constant for G_1-to-S/G_2 transition
k_8	10^6 min^{-1}	rate constant for S/G_2-to-M transition
k_9	10^6 min^{-1}	rate constant for M-to-G_1 transition
t_7	250 molecules	threshold for G_1-to-S/G_2 transition
t_8	70 molecules	threshold for S/G_2-to-M transition
t_9	20 molecules	threshold for M-to-G_1 transition
$init_C$	100 cells	initial cell amount
k_{death}	0.006 min^{-1}	rate constant for cell death

3 Single-cell model of cell cycle regulation and pheromone response (Figure 7B-D)

3.1 Species names

- fission yeast cell: C with $ar(C) = 2$
- unphosphorylated cyclin: Y with $ar(Y) = 0$
- phosphorylated cyclin: Y_P with $ar(Y_P) = 0$
- cdc2: D with $ar(D) = 0$
- inactive MPF: M_I with $ar(M_I) = 0$
- active MPF: M_A with $ar(M_A) = 0$
- repressed MPF: M_R with $ar(M_R) = 0$
- pheromone (P-/M-factor): F with $ar(F) = 0$

3.2 Rule schemata

Cyclin synthesis and formation of inactive MPF complex (1-2)

- $C(v,p)[s]^c \xrightarrow{k_1 \cdot c} C(v,p)[Y + s]$
- $Y^y + D^d \xrightarrow{k_2 \cdot y \cdot d} M_I$

MPF activation (3)

- $M_I^i + M_A^a \xrightarrow{(k_3' + k_3(a/D_{tot})^2)i} 2\,M_A$

Breakage of activated MPF complex and cyclin degradation (4-5)

- $C(v,p)[M_A^a + s]^c \xrightarrow[a>1]{(k_4/v)a \cdot c} C(v,p)[Y_P + D + s]$
- $Y_P^y \xrightarrow{k_5 \cdot y} \emptyset$

Cell growth (6)

- $C(v,p)[s]^c \xrightarrow[p \in \{\mathbf{G1}, \mathbf{SG2}\}]{k_6 \cdot c} C(v + (1/T_d), p)[s]$

Cell cycle transitions (7-8)

- $C(v, \mathbf{G1})[M_I^i + s]^c \xrightarrow[i>t_7]{k_7 \cdot c} C(v, \mathbf{SG2})[M_I + s]$
- $C(v, \mathbf{SG2})[M_A^a + s]^c \xrightarrow[a>t_8]{k_8 \cdot c} C(v, \mathbf{M})[M_A + s]$

Cell division (9)

- $C(v, \mathbf{M})[M_A^a + s]^c \xrightarrow[a<t_9]{k_9 \cdot c} C(\frac{v}{2}, \mathbf{G1})[M_A + s]$

Pheromone induced MPF inhibition (11-12)

- $C(v,p)[M_I^i + s]^c + F^f \xrightarrow{(H/v^2)\cdot i\cdot c} C(v,p)[M_R + s] + F$
  with $H = \frac{k_{11}\cdot f^3}{K_{11}^3 + f^3}$

- $M_R^r \xrightarrow{k_{12}\cdot r} M_I$

3.3 Initial solution

$init_F\, F + C(1.0, \mathbf{G1})[(D_{tot} - 1)\, D + M_A]$

3.4 Parameters

Parameter	Value	Description
D_{tot}	1000 molecules	total amount of cdc2 molecules
T_d	232 min	mass-doubling time
k_1	$0.015 \cdot D_{tot}$ min^{-1}	rate constant for cyclin synthesis
k_2	200 min^{-1}	rate constant for inactive MPF formation
k_3'	0.018 min^{-1}	rate constant for basal MPF activation
k_3	180 min^{-1}	rate constant for MPF activation
k_4	4.5 min^{-1}	rate constant for MPF dissociation
k_5	0.6 min^{-1}	rate constant for degradation of phosphorylated cyclin
k_6	1.0 min^{-1}	rate constant for cell growth
k_7	10^6 min^{-1}	rate constant for G_1-to-S/G_2 transition
k_8	10^6 min^{-1}	rate constant for S/G_2-to-M transition
k_9	10^6 min^{-1}	rate constant for M-to-G_1 transition
k_{11}	1.5 min^{-1}	rate constant for MPF inhibition
K_{11}	800 molecules	pheromone amount at which rate is half of the maximal rate
k_{12}	0.02 min^{-1}	rate constant for MPF recovery
t_7	250 molecules	threshold for G_1-to-S/G_2 transition
t_8	70 molecules	threshold for S/G_2-to-M transition
t_9	20 molecules	threshold for M-to-G_1 transition
$init_F$	$0 - 600$ molecules	extracellular pheromone amount

4 Multi-cellular model extended by pheromone secretion and response (Figure 8B)

4.1 Species names

- fission yeast cell: C with $ar(C) = 4$
- unphosphorylated cyclin: Y with $ar(Y) = 0$
- phosphorylated cyclin: Y_P with $ar(Y_P) = 0$
- cdc2: D with $ar(D) = 0$
- inactive MPF: M_I with $ar(M_I) = 0$
- active MPF: M_A with $ar(M_A) = 0$
- repressed MPF: M_R with $ar(M_R) = 0$
- P-factor pheromone: F_P with $ar(F_P) = 0$
- M-factor pheromone: F_M with $ar(F_M) = 0$
- Sxa2: X with $ar(X) = 0$

4.2 Rule schemata

Cyclin synthesis and formation of inactive MPF complex (1-2)

- $C(v, p, t, w)[s]^c \xrightarrow{k_1 \cdot c} C(v, p, t, w)[Y + s]$
- $Y^y + D^d \xrightarrow{k_2 \cdot y \cdot d} M_I$

MPF activation (3)

- $M_I^i + M_A^a \xrightarrow{(k_3' + k_3 (a/D_{tot})^2) i} 2\, M_A$

Breakage of activated MPF complex and cyclin degradation (4-5)

- $C(v, p, t, w)[M_A^a + s]^c \xrightarrow[a>1]{(k_4/v) a \cdot c} C(v, p, t, w)[Y_P + D + s]$
- $Y_P^y \xrightarrow{k_5 \cdot y} \emptyset$

Cell growth (6)

- $C(v, p, t, w)[s]^c \xrightarrow[p \in \{\mathbf{G1}, \mathbf{SG2}\}]{k_6 \cdot c} C(v + (1/T_d), p, t, w)[s]$

Cell cycle transitions (7-8)

- $C(v, \mathbf{G1}, t, w)[M_I^i + s]^c \xrightarrow[i>t_7]{k_7 \cdot c} C(v, \mathbf{SG2}, t, w)[M_I + s]$
- $C(v, \mathbf{SG2}, t, w)[M_A^a + s]^c \xrightarrow[a>t_8]{k_8 \cdot c} C(v, \mathbf{M}, t, w)[M_A + s]$

Cell division (9)

- $C(v, \mathbf{M}, t, \mathbf{U})[M_A^a + s]^c \xrightarrow[a<t_9]{k_9 \cdot c}$
  $C(\frac{v}{2}, \mathbf{G1}, t, \mathbf{S})[M_A + s] + C(\frac{v}{2}, \mathbf{G1}, t, \mathbf{U})[M_A + s]$

- $C(v, \mathbf{M}, t, \mathbf{S})[M_A^a + s]^c \xrightarrow[a<t_9]{k_9 \cdot c}$
  $C(\frac{v}{2}, \mathbf{G1}, t, \mathbf{S})[M_A + s] + C(\frac{v}{2}, \mathbf{G1}, \texttt{if } t = \mathbf{P} \texttt{ then } \mathbf{M} \texttt{ else } \mathbf{P}, \mathbf{U})[M_A + s]$

Pheromone secretion (10)

- $C(v, p, \mathbf{P}, w)[s]^c \xrightarrow{k_{10} \cdot c} C(v, p, \mathbf{P}, w)[s] + F_P$

- $C(v, p, \mathbf{M}, w)[s]^c \xrightarrow{k_{10} \cdot c} C(v, p, \mathbf{M}, w)[s] + F_M$

Pheromone induced MPF inhibition (11-12)

- $C(v, p, \mathbf{M}, w)[M_I^i + s]^c + F_P^f \xrightarrow{(H/v^2) \cdot i \cdot c} C(v, p, \mathbf{M}, w)[M_R + s] + F_P$
  - where $H = \frac{k_{11} \cdot f^3}{K_{11}^3 + f^3}$

- $C(v, p, \mathbf{P}, w)[M_I^i + s]^c + F_M^f \xrightarrow{(H/v^2) \cdot i \cdot c} C(v, p, \mathbf{P}, w)[M_R + s] + F_M$
  - where $H = \frac{k_{11} \cdot f^3}{K_{11}^3 + f^3}$

- $M_R^r \xrightarrow{k_{12} \cdot r} M_I$

Pheromone degradation (diffusion out of system) (13)

- $F_P^f \xrightarrow{k_{13} \cdot f} \emptyset$

- $F_M^f \xrightarrow{k_{13} \cdot f} \emptyset$

Sxa2 turnover and catalytic P-factor degradation (10,13,15)

- $C(v, p, \mathbf{M}, w)[s]^c \xrightarrow{(k_{10}/10) \cdot c} C(v, p, \mathbf{M}, w)[s] + X$

- $X^x \xrightarrow{k_{13} \cdot x} \emptyset$

- $X^x + F_P^p \xrightarrow{k_{15} \cdot x \cdot p} X$

4.3 Initial solution

$C(1.0, \mathbf{G1}, \mathbf{P}, \mathbf{U})[(D_{tot} - 1)\, D + M_A] + C(1.0, \mathbf{G1}, \mathbf{M}, \mathbf{U})[(D_{tot} - 1)\, D + M_A]$

4.4 Parameters

Parameter	Value	Description
D_{tot}	1000 molecules	total amount of cdc2 molecules
T_d	232 min	mass-doubling time
k_1	$0.015 \cdot D_{tot}$ min^{-1}	rate constant for cyclin synthesis
k_2	200 min^{-1}	rate constant for inactive MPF formation
k'_3	0.018 min^{-1}	rate constant for basal MPF activation
k_3	180 min^{-1}	rate constant for MPF activation
k_4	4.5 min^{-1}	rate constant for MPF dissociation
k_5	0.6 min^{-1}	rate constant for degradation of phosphorylated cyclin
k_6	1.0 min^{-1}	rate constant for cell growth
k_7	10^6 min^{-1}	rate constant for G_1-to-S/G_2 transition
k_8	10^6 min^{-1}	rate constant for S/G_2-to-M transition
k_9	10^6 min^{-1}	rate constant for M-to-G_1 transition
k_{10}	1.0 min^{-1}	rate constant for pheromone production
k_{11}	1.5 min^{-1}	rate constant for MPF inhibition
K_{11}	800 molecules	pheromone amount at which rate is half of the maximal rate
k_{12}	0.02 min^{-1}	rate constant for MPF recovery
k_{13}	0.005 min^{-1}	rate constant for pheromone/Sxa2 degradation
k_{15}	10^{-4} min^{-1}	rate constant for catalytic P-factor degradation
t_7	250 molecules	threshold for G_1-to-S/G_2 transition
t_8	70 molecules	threshold for S/G_2-to-M transition
t_9	20 molecules	threshold for M-to-G_1 transition

5 Entire model comprising of additional spatial dynamics

5.1 Species names

- fission yeast cell: C with $ar(C) = 4$
- unphosphorylated cyclin: Y with $ar(Y) = 0$
- phosphorylated cyclin: Y_P with $ar(Y_P) = 0$
- cdc2: D with $ar(D) = 0$
- inactive MPF: M_I with $ar(M_I) = 0$
- active MPF: M_A with $ar(M_A) = 0$
- repressed MPF: M_R with $ar(M_R) = 0$
- P-factor pheromone: F_P with $ar(F_P) = 0$
- M-factor pheromone: F_M with $ar(F_M) = 0$
- Sxa2: X with $ar(X) = 0$
- voxel of the grid: G with $ar(G) = 3$

5.2 Rule schemata

Cyclin synthesis and formation of inactive MPF complex (1-2)

- $C(v, p, t, w)[s]^c \xrightarrow{k_1 \cdot c} C(v, p, t, w)[Y + s]$
- $Y^y + D^d \xrightarrow{k_2 \cdot y \cdot d} M_I$

MPF activation (3)

- $M_I^i + M_A^a \xrightarrow{(k_3' + k_3(a/D_{tot})^2)i} 2\, M_A$

Breakage of activated MPF complex and cyclin degradation (4-5)

- $C(v, p, t, w)[M_A^a + s]^c \xrightarrow[a>1]{(k_4/v)a \cdot c} C(v, p, t, w)[Y_P + D + s]$
- $Y_P^y \xrightarrow{k_5 \cdot y} \emptyset$

Cell growth (6)

- $C(v, p, t, w)[s]^c \xrightarrow[p \in \{\mathbf{G1}, \mathbf{SG2}\}]{k_6 \cdot c} C(v + (1/T_d), p, t, w)[s]$

Cell cycle transitions (7-8)

- $C(v, \mathbf{G1}, t, w)[M_I^i + s]^c \xrightarrow[i>t_7]{k_7 \cdot c} C(v, \mathbf{SG2}, t, w)[M_I + s]$
- $C(v, \mathbf{SG2}, t, w)[M_A^a + s]^c \xrightarrow[a>t_8]{k_8 \cdot c} C(v, \mathbf{M}, t, w)[M_A + s]$

Cell division (9)

- $G(x,y,n)[C(v,\mathbf{M},t,\mathbf{U})[M_A^a + s_C]^c + s_G] \xrightarrow[a<t_9]{k_9 \cdot c}$
  $G(x,y,n+1)[C(\frac{v}{2},\mathbf{G1},t,\mathbf{S})[M_A + s_C] + C(\frac{v}{2},\mathbf{G1},t,\mathbf{U})[M_A + s_C] + s_G]$

- $G(x,y,n)[C(v,\mathbf{M},t,\mathbf{S})[M_A^a + s_C]^c + s_G] \xrightarrow[a<t_9]{k_9 \cdot c}$
  $G(x,y,n{+}1)[C(\frac{v}{2},\mathbf{G1},t,\mathbf{S})[M_A{+}s_C]{+}C(\frac{v}{2},\mathbf{G1},\texttt{if}\ t=\mathbf{P}\ \texttt{then}\ \mathbf{M}\ \texttt{else}\ \mathbf{P},\mathbf{U})[M_A{+}s_C]{+}s_G]$

Pheromone secretion (10)

- $C(v,p,\mathbf{P},w)[s]^c \xrightarrow{k_{10} \cdot c} C(v,p,\mathbf{P},w)[s] + F_P$

- $C(v,p,\mathbf{M},w)[s]^c \xrightarrow{k_{10} \cdot c} C(v,p,\mathbf{M},w)[s] + F_M$

Pheromone induced MPF inhibition (11-12)

- $C(v,p,\mathbf{M},w)[M_I^i + s]^c + F_P^f \xrightarrow{(H/v^2) \cdot i \cdot c} C(v,p,\mathbf{M},w)[M_R + s] + F_P$
  - where $H = \frac{k_{11} \cdot f^3}{K_{11}^3 + f^3}$

- $C(v,p,\mathbf{P},w)[M_I^i + s]^c + F_M^f \xrightarrow{(H/v^2) \cdot i \cdot c} C(v,p,\mathbf{P},w)[M_R + s] + F_M$
  - where $H = \frac{k_{11} \cdot f^3}{K_{11}^3 + f^3}$

- $M_R^r \xrightarrow{k_{12} \cdot r} M_I$

Sxa2 turnover and catalytic P-factor degradation (10,13,15)

- $C(v,p,\mathbf{M},w)[s]^c \xrightarrow{(k_{10}/10) \cdot c} C(v,p,\mathbf{M},w)[s] + X$

- $X^x \xrightarrow{k_{13} \cdot x} \emptyset$

- $X^x + F_P^p \xrightarrow{k_{15} \cdot x \cdot p} X$

Pheromone diffusion (13)

- $G(x_1,y_1,n_1)[F_P^f + s_1] + G(x_2,y_2,n_2)[s_2] \xrightarrow[nb(x_1,y_1,x_2,y_2)]{(k_{13}/4) \cdot f} G(x_1,y_1,n_1)[s_1] + G(x_2,y_2,n_2)[F_P + s_2]$

- $G(x,y,n)[F_P^f + s] \xrightarrow[bor_x(x)]{(k_{13}/4) \cdot f} G(x,y,n)[s]$

- $G(x,y,n)[F_P^f + s] \xrightarrow[bor_y(y)]{(k_{13}/4) \cdot f} G(x,y,n)[s]$

- $G(x_1,y_1,n_1)[F_M^f + s_1] + G(x_2,y_2,n_2)[s_2] \xrightarrow[nb(x_1,y_1,x_2,y_2)]{(k_{13}/4) \cdot f} G(x_1,y_1,n_1)[s_1] + G(x_2,y_2,n_2)[F_M + s_2]$

- $G(x,y,n)[F_M^f + s] \xrightarrow[bor_x(x)]{(k_{13}/4) \cdot f} G(x,y,n)[s]$

- $G(x,y,n)[F_M^f + s] \xrightarrow[bor_y(y)]{(k_{13}/4)\cdot f} G(x,y,n)[s]$
- with functions
  - $nb(x_1,y_1,x_2,y_2) =$ `if` $(x_1 = x_2 \wedge (y_1 = y_2 + 1 \vee y_1 = y_2 - 1)) \vee (y_1 = y_2 \wedge (x_1 = x_2 + 1 \vee x_1 = x_2 - 1))$ `then` **true** `else` **false**
  - $bor_x(x) =$ `if` $x = 1 \vee x = x_{max}$ `then` **true** `else` **false**
  - $bor_y(y) =$ `if` $y = 1 \vee y = y_{max}$ `then` **true** `else` **false**

Displacement of cells from crowded locations (14)

- $G(x_1,y_1,n_1)[C(v,p,t,w)[s_C] + s_1] + G(x_2,y_2,n_2)[s_2] \xrightarrow[nb(x_1,y_1,x_2,y_2),n_1>4]{k_{14}\frac{n_1^2}{1+n_2^2}}$ $G(x_1,y_1,n_1-1)[s_1] + G(x_2,y_2,n_2+1)[C(v,p,t,w)[s_C] + s_2]$
- with function
  - $nb(x_1,y_1,x_2,y_2) =$ `if` $(x_1 = x_2 \wedge (y_1 = y_2 + 1 \vee y_1 = y_2 - 1)) \vee (y_1 = y_2 \wedge (x_1 = x_2 + 1 \vee x_1 = x_2 - 1))$ `then` **true** `else` **false**

5.3 Initial solution

$G(3,3,2)[C(1.0,\mathbf{G1},\mathbf{P},\mathbf{U})[(D_{tot}-1)\,D+M_A]+C(1.0,\mathbf{G1},\mathbf{M},\mathbf{U})[(D_{tot}-1)\,D+M_A]]+remaining\ Gs$ where $remaining\ Gs = \{G(1,1,0) + ... + G(x_{max},y_{max},0)\}\backslash G(3,3,n)$

5.4 Parameters

Parameter	Value	Description
D_{tot}	1000 molecules	total amount of cdc2 molecules
T_d	232 min	mass-doubling time
k_1	$0.015 \cdot D_{tot}$ min^{-1}	rate constant for cyclin synthesis
k_2	200 min^{-1}	rate constant for inactive MPF formation
k_3'	0.018 min^{-1}	rate constant for basal MPF activation
k_3	180 min^{-1}	rate constant for MPF activation
k_4	4.5 min^{-1}	rate constant for MPF dissociation
k_5	0.6 min^{-1}	rate constant for degradation of phosphorylated cyclin
k_6	1.0 min^{-1}	rate constant for cell growth
k_7	10^6 min^{-1}	rate constant for G_1-to-S/G_2 transition
k_8	10^6 min^{-1}	rate constant for S/G_2-to-M transition
k_9	10^6 min^{-1}	rate constant for M-to-G_1 transition
k_{10}	1.0 min^{-1}	rate constant for pheromone production
k_{11}	1.5 min^{-1}	rate constant for MPF inhibition
K_{11}	800 molecules	pheromone amount at which rate is half of the maximal rate
k_{12}	0.02 min^{-1}	rate constant for MPF recovery
k_{13}	0.005 min^{-1}	rate constant for pheromone diffusion/Sxa2 degradation
k_{14}	1.0 min^{-1}	rate constant for cell movement
k_{15}	10^{-4} min^{-1}	rate constant for catalytic P-factor degradation
t_7	250 molecules	threshold for G_1-to-S/G_2 transition
t_8	70 molecules	threshold for S/G_2-to-M transition
t_9	20 molecules	threshold for M-to-G_1 transition
x_{max}	5	number of voxels in the x-dimension
y_{max}	5	number of voxels in the y-dimension

5.5 Simulation results

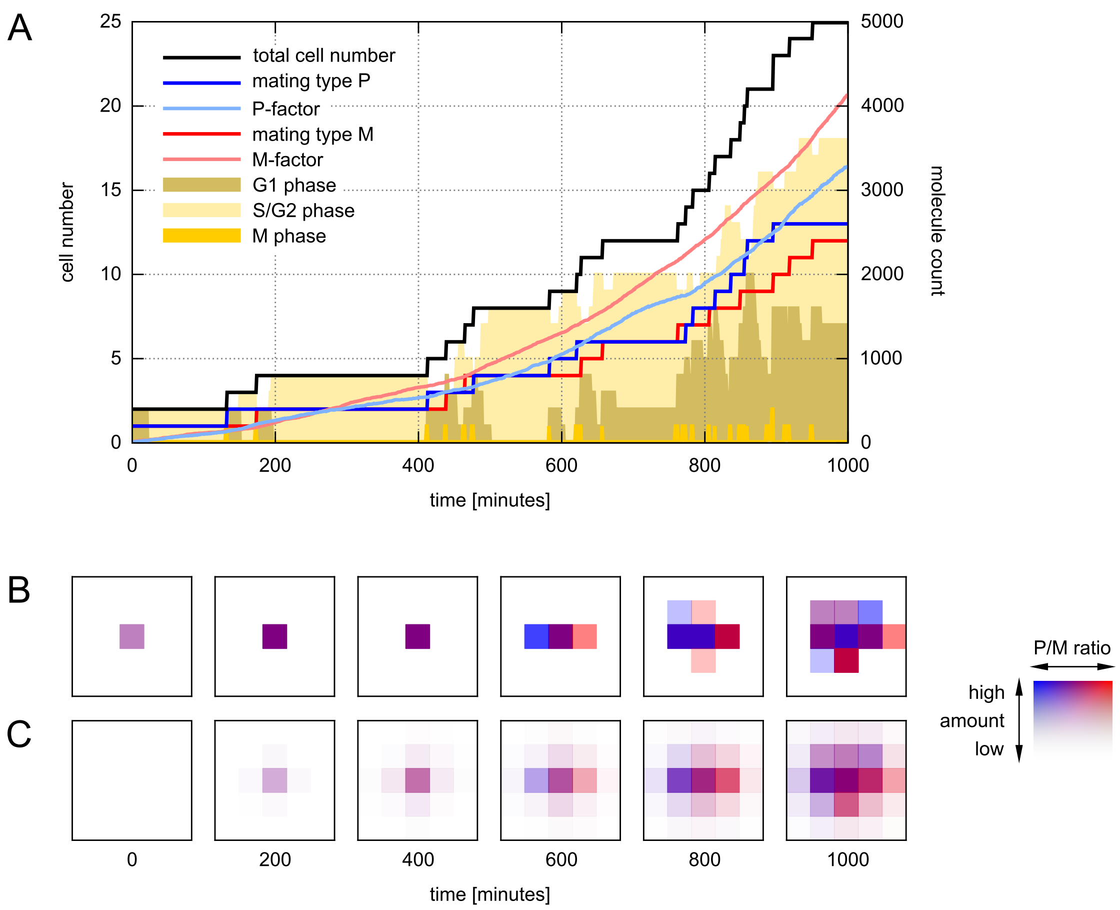

Figure 1: Simulation results of the entire model including the spatial layer. A 5×5 matrix defines the neighborhood of the grid-voxels. Mass-doubling time $T_d = 232$ min. (A) Trajectories of the overall cell population and pheromone molecules. After 1000 minutes one third of all cells are arrested in the G_1 phase of the cell cycle. (B) Heatmap of the spatial distribution of cells at six distinct time points. The ratio between cells of mating type P and M is depicted by the color tone, the total amount of cells per grid-volume by the color intensity. (C) Heatmap of the spatial distribution of pheromone molecules. The color tone and intensity denote the ratio between P-factor and M-factor molecules and the total pheromone amount respectively.
